# Proteomic Landscape of Spermatozoa Under Inflammatory and Oxidative Injury: Molecular Mechanisms and Clinical Translation

**DOI:** 10.3390/antiox15091192

**Published:** 2026-09-18

**Authors:** Emanuela Teveroni, Michela Cicchinelli, Edoardo Vergani, Salvatore Raia, Alfredo Pontecorvi, Andrea Urbani, Domenico Milardi, Federica Iavarone

**Affiliations:** 1 Unit of Chemistry, Biochemistry and Molecular Biology, Fondazione Policlinico Universitario A. Gemelli IRCCS, Largo A. Gemelli 1, 00168 Rome, Italy; emanuela.teveroni@guest.policlinicogemelli.it (E.T.); michela.cicchinelli@unicatt.it (M.C.); andrea.urbani@policlinicogemelli.it (A.U.); federica.iavarone@unicatt.it (F.I.); 2 Università Cattolica del Sacro Cuore, Largo F. Vito 1, 00168 Rome, Italy; edoardo.vergani@guest.policlinicogemelli.it (E.V.); alfredo.pontecorvi@policlinicogemelli.it (A.P.); 3 Department of Basic Biotechnological Sciences, Intensive and Perioperative Clinics, Università Cattolica del Sacro Cuore, Largo F. Vito 1, 00168 Rome, Italy; 4 Complex Operative Unit of Internal Medicine, Endocrinology and Diabetology, Department of Translational Medicine and Surgery, Fondazione Policlinico Universitario A. Gemelli IRCCS, Largo A. Gemelli 1, 00168 Rome, Italy; salvatore.raia@guest.policlinicogemelli.it

**Keywords:** male infertility, sperm proteomics, oxidative stress, inflammation, seminal plasma biomarkers, mass spectrometry

## Abstract

Male infertility is a multifactorial condition in which conventional semen analysis provides limited insight into the molecular mechanisms underlying sperm dysfunction. Inflammatory and oxidative processes are major contributors to impaired male reproductive function, acting through interconnected alterations in redox homeostasis, immune activation, and protein integrity. Because mature spermatozoa have minimal transcriptional and translational capacity, oxidative and inflammatory damage to proteins may persist and substantially affect motility, capacitation, fertilization competence, and reproductive outcomes. This review examines how sperm proteomics can characterize the molecular landscape of inflammatory and oxidative injury in male infertility. Particular attention is given to pre-analytical variables, sperm purification, protein extraction, bottom-up LC–MS/MS workflows, data-dependent and data-independent acquisition, quantitative strategies, and targeted or orthogonal validation. Proteomic evidence identifies coordinated remodeling of flagellar and motility proteins, Ca^2+^-signaling networks, mitochondrial bioenergetics, antioxidant defenses, membrane-associated pathways, and proteins involved in sperm–oocyte interaction. These signatures may improve mechanistic patient stratification and support the development of biomarker panels linked to clinically relevant endpoints. Nevertheless, routine implementation remains limited by methodological heterogeneity, inadequate standardization, small study populations, and insufficient prospective validation. Integrating proteomic signatures with redox, inflammatory, clinical, and reproductive outcome data may facilitate the transition from descriptive semen assessment and empirical treatment toward mechanism-based precision andrology.

## 1. Introduction

Male infertility is increasingly recognized as a multifactorial condition in which classical semen parameters only partially capture the underlying biological complexity. Beyond alterations in sperm concentration, motility, and morphology, a growing body of evidence indicates that inflammatory and oxidative mechanisms play a central role in the impairment of sperm function and fertilizing competence. Reactive oxygen species (ROS) are physiologically involved in sperm maturation and function, including capacitation, hyperactivation, acrosome reaction and sperm–oocyte interaction [1]. However, when ROS generation exceeds the antioxidant buffering capacity of the seminal environment, this finely regulated redox signaling is converted into oxidative injury, with detrimental effects on sperm membranes, proteins, mitochondria and DNA integrity [1,2,3,4]. Inflammation represents one of the major biological contexts in which this redox imbalance may arise. Along the male reproductive tract, inflammatory activation can involve the testis, epididymis, and male accessory glands, including the prostate, seminal vesicles, and bulbourethral glands, leading to changes in the cellular and biochemical composition of semen. Activated leukocytes, epithelial cells, and damaged or immature germ cells may contribute to increased ROS generation. In parallel, inflammatory cytokines and altered glandular secretions can further compromise the antioxidant defenses normally provided by seminal plasma [5]. This creates a self-amplifying loop in which inflammation promotes oxidative stress, and oxidative stress in turn sustains tissue damage, immune activation and sperm dysfunction [5,6,7]. In this landscape, spermatozoa are not passive targets of a hostile microenvironment, but highly specialized cells whose functional competence is particularly dependent on the preservation of membrane architecture, mitochondrial activity, cytoskeletal organization and tightly regulated redox-sensitive signaling pathways. Because mature spermatozoa have limited transcriptional and translational activity, oxidative and inflammatory insults cannot be efficiently compensated by de novo protein synthesis or extensive repair mechanisms. Therefore, damage to sperm proteins and redox-sensitive pathways may have a disproportionate impact on motility, capacitation, fertilization competence and embryo developmental potential [8,9,10]. Proteomic approaches provide a powerful framework to investigate these mechanisms, as they allow the identification of coordinated changes in sperm proteins and pathways that are not captured by conventional semen analysis. This is especially relevant in inflammatory and oxidative conditions, where the sperm proteome may act both as a molecular readout of damage and as a source of candidate biomarkers for clinical translation [4,9].

### 1.1. Inflammation–Oxidative Stress Crosstalk Along the Male Reproductive Tract

The male reproductive tract is particularly exposed to the biological consequences of inflammation-driven oxidative stress. Inflammatory conditions affecting the testis, epididymis, prostate and accessory glands may alter both the production and the composition of seminal plasma, thereby modifying the microenvironment in which spermatozoa acquire, maintain and express their functional competence. Seminal plasma normally provides antioxidant protection, energy substrates, regulatory proteins and signaling molecules that support sperm survival and function after ejaculation. When inflammation disrupts this balance, spermatozoa may be exposed to increased concentrations of ROS, cytokines, proteases, lipid mediators and other inflammatory products, with direct consequences on membrane integrity, motility and fertilizing potential [5,6]. A central mechanism linking inflammation to oxidative injury is the activation of leukocytes within semen. Neutrophils and macrophages are among the most relevant ROS-producing cells in the male genital tract, especially during infection or sterile inflammation. Through enzymatic systems such as NADPH oxidase and myeloperoxidase-dependent pathways, activated leukocytes can generate high levels of superoxide anion, hydrogen peroxide and downstream reactive species. Although controlled ROS production is compatible with physiological sperm signaling, excessive leukocyte-derived ROS may overwhelm seminal antioxidant defenses and initiate lipid peroxidation, protein oxidation and DNA damage [4,5,11]. This process is not limited to infectious conditions. Non-bacterial or chronic inflammatory states may also generate a pro-oxidant seminal milieu through persistent immune activation, altered epithelial secretory function and recruitment of inflammatory mediators. In the prostate and accessory glands, inflammation may modify the concentration of antioxidants, metal-binding proteins, enzymes, extracellular vesicles and immune-related proteins released into seminal plasma. Since spermatozoa are highly dependent on this extracellular support, changes in seminal plasma composition may translate into impaired motility, defective capacitation, altered membrane remodeling and reduced fertilization competence. Thus, the inflammatory status of the male accessory glands can influence sperm function even in the absence of overt infection. In this context, redox and inflammatory biomarkers, together with molecular profiling approaches, may improve the characterization of patients with inflammation-related sperm dysfunction.

### 1.2. Why Spermatozoa Are Uniquely Vulnerable to Redox Imbalance

Spermatozoa are uniquely vulnerable to redox imbalance because their structural and functional specialization is achieved at the expense of several protective mechanisms that are common in somatic cells. Mature spermatozoa are transcriptionally and translationally limited, contain very little cytoplasm and have a highly specialized plasma membrane enriched in polyunsaturated fatty acids. These features are essential for sperm motility, membrane fluidity, capacitation and sperm–oocyte fusion, but they also make spermatozoa particularly sensitive to oxidative injury. The high content of polyunsaturated fatty acids provides abundant substrates for lipid peroxidation, while the reduced cytoplasmic volume limits the intracellular availability of antioxidant enzymes and low-molecular-weight scavengers [10,11,12]. Lipid peroxidation is one of the main mechanisms through which oxidative stress impairs sperm function. Once initiated, peroxidation of membrane lipids can propagate as a chain reaction, generating reactive aldehydes and secondary oxidation products that alter membrane fluidity, ion transport, mitochondrial function and axonemal activity. Since sperm motility depends on the coordinated integrity of the plasma membrane, flagellar cytoskeleton, ion channels and mitochondrial energy metabolism, lipid peroxidation provides a direct mechanistic link between oxidative stress and reduced progressive motility. This is particularly relevant in inflammatory conditions, where extracellular ROS production may continuously expose spermatozoa to a pro-oxidant environment [8,10,11]. Another major determinant of sperm redox sensitivity is the narrow physiological window in which ROS exert beneficial versus detrimental effects. Low and tightly regulated ROS levels are required for key sperm functions, including capacitation, cholesterol efflux, cAMP-dependent signaling, protein tyrosine phosphorylation, hyperactivation and acrosome reaction. Therefore, ROS are not merely toxic by-products, but physiological signaling molecules. However, when ROS exceed the antioxidant buffering capacity of spermatozoa and seminal plasma, redox signaling is converted into oxidative damage. This dual role explains why male reproductive biology should be interpreted in terms of redox balance rather than simple ROS suppression [1,3,13]. Mitochondria further contribute to the vulnerability of spermatozoa to redox imbalance. Located in the midpiece, sperm mitochondria support energy production and participate in redox-regulated signaling processes required for motility. At the same time, dysfunctional mitochondria can become a major source of ROS, establishing a vicious cycle in which oxidative stress impairs mitochondrial activity and mitochondrial dysfunction further increases ROS generation. Because flagellar movement requires both ATP availability and preserved axonemal architecture, mitochondrial oxidative damage may rapidly translate into reduced motility and fertilizing competence.

Sperm proteins are also major targets of oxidative and inflammatory injury. Redox imbalance can induce carbonylation, thiol oxidation, S-nitrosylation, S-glutathionylation, disulfide bond rearrangements and other redox-dependent post-translational modifications [9,14]. These changes may affect enzymes, structural proteins, chaperones, mitochondrial proteins, ion channels and proteins involved in capacitation or sperm–egg interaction. However, altered protein abundance and oxidative post-translational modification should be considered as distinct, although potentially convergent, molecular events. Conventional shotgun proteomics mainly identifies differences in protein abundance, whereas the direct demonstration of oxidative modification requires dedicated redox-proteomic or PTM-targeted approaches. Therefore, reduced abundance of a sperm protein should not be automatically interpreted as oxidative inactivation unless specific evidence of carbonylation, thiol oxidation, S-nitrosylation, S-glutathionylation, tyrosine nitration, disulfide rearrangement or other redox-dependent modifications is available [9,14].

Several examples illustrate this distinction. In the redox-defense module, peroxiredoxins are direct redox-sensitive targets in spermatozoa. PRDX6 is particularly relevant because it contributes to antioxidant protection and lipid peroxide detoxification in human spermatozoa. PRDX6 dysfunction may arise through mechanistically different processes, including reduced protein abundance or direct oxidative modification of its catalytic cysteine residue, with the latter potentially impairing its peroxidase activity. Thus, reduced PRDX6 abundance and oxidative inactivation of PRDX6 should not be used interchangeably, even though both may converge on impaired redox homeostasis and reduced sperm function [15].

In the capacitation and signaling module, redox-dependent PTMs may also have physiological rather than exclusively pathological significance. Nitric oxide-related pathways can modulate sperm function through S-nitrosylation of multiple sperm proteins, including proteins involved in metabolism, chaperone activity, cytoskeletal organization and sperm–egg interaction. Similarly, controlled ROS levels participate in phosphorylation-dependent events during capacitation. Therefore, redox modifications may contribute to normal sperm signaling when tightly regulated, whereas excessive or misplaced modifications may become detrimental under oxidative stress conditions [1,3,13,16].

In mature spermatozoa, where protein turnover and repair are extremely limited, both mechanisms may have relevant functional consequences. Abundance changes may reflect defective spermatogenesis, altered epididymal maturation, selective loss of sperm subpopulations or treatment-associated remodeling. By contrast, oxidative PTMs may directly alter catalytic activity, protein–protein interactions, localization or structural stability. For this reason, oxidative stress should not be considered only as a source of lipid or DNA damage, but also as a driver of sperm protein dysfunction and, in selected contexts, proteomic remodeling with potential consequences for sperm function and clinical reproductive outcomes [4,9,14].

Taken together, these features explain why spermatozoa are exceptionally sensitive to inflammatory and oxidative injury. The same biological properties that make them efficient fertilizing cells—membrane fluidity, mitochondrial specialization, compact morphology, reduced cytoplasm and redox-dependent signaling—also create a condition of intrinsic vulnerability. In inflammatory male infertility, this vulnerability becomes clinically relevant because the seminal environment may shift from a supportive medium to a source of oxidative and proteomic damage. This provides the rationale for using sperm proteomics to identify molecular signatures of inflammatory and oxidative injury, provided that abundance-based proteomic findings are clearly distinguished from direct evidence of oxidative post-translational modification.

## 2. Operational Definitions: Inflammatory and Oxidative Injury in Male Infertility

Inflammatory and oxidative injury in male infertility should be regarded as interconnected but not interchangeable biological conditions. Inflammation refers to the activation of immune, epithelial and stromal responses within the male reproductive tract, often characterized by leukocyte recruitment, cytokine release, altered glandular secretion and remodeling of the seminal microenvironment. Oxidative injury, in contrast, reflects an imbalance between reactive oxygen species production and antioxidant defenses, resulting in damage to sperm lipids, proteins, mitochondria and DNA. Although these processes frequently coexist, their relative contribution may vary across clinical phenotypes, explaining why patients with similar semen parameters may display different molecular signatures and reproductive outcomes [4,17,18]. In clinical practice and translational research, it is therefore useful to define inflammatory–oxidative sperm injury as a spectrum rather than a single diagnostic entity. At one end, inflammation-dominant conditions may be characterized by increased cytokines, leukocytospermia or accessory gland dysfunction, with oxidative stress acting as a secondary mediator. Conversely, oxidative stress-dominant conditions may arise from varicocele, metabolic disease, environmental exposures or aging, with inflammation contributing as a systemic or local amplifier. Between these extremes, most infertile men probably exhibit mixed phenotypes, in which inflammatory and oxidative mechanisms converge on common sperm targets, including membrane integrity, motility-related structures, mitochondrial function, chromatin stability and redox-sensitive protein networks [1,10,11].

This operational distinction is particularly relevant for proteomic studies. Conventional semen analysis provides functional and morphological endpoints, but it cannot determine whether sperm dysfunction reflects active inflammation, oxidative damage, impaired antioxidant buffering, defective spermatogenesis, accessory gland dysfunction or combinations of these mechanisms. Proteomics can help bridge this gap by revealing pathway-level alterations in mitochondrial metabolism, cytoskeletal organization, flagellar function, redox homeostasis, immune activation, extracellular vesicle signaling and sperm–egg interaction. Thus, defining inflammatory and oxidative injury at the biochemical and molecular level is essential to improve patient stratification and to avoid analyzing biologically heterogeneous groups as if they represented a single disease process [4,9].

### 2.1. Clinical Contexts: MAGI/Prostatitis, Varicocele, Metabolic, Environmental and Aging-Related Injury

Male accessory gland inflammation/infection (MAGI) represents a highly prevalent (2–18% men) and clinically relevant model of inflammation-driven semen alteration that can frequently lead to infertility and sexual dysfunction [19,20,21]. This wide range may partly reflect substantial heterogeneity in MAGI definitions, diagnostic criteria, microbiological assessment, ultrasound-based phenotyping and inclusion of symptomatic versus asymptomatic patients, underscoring the risk of misclassification in studies of inflammatory male infertility [21].

MAGI refers to inflammatory disorders involving the prostate, seminal vesicles, epididymis and sometimes related excurrent ducts; the term encompasses a wide spectrum of both infectious and non-infectious conditions [21,22]. The diagnosis, according to the WHO 1993 Algorithm, is a combination of abnormal semen parameters and history of genitourinary infection or physical signs and/or abnormal prostatic fluid or urine after prostatic massage and/or leucocytospermia or modified semen rheological parameters [21]. Ultrasound can support clinical diagnosis and stage the type/extent of disease [23,24]. Inflammation involving male accessory glands may modify seminal plasma composition and expose spermatozoa to cytokines, leukocyte-derived oxidants, proteases and altered secretory products. In MAGI, this may translate into impaired motility, altered viscosity or liquefaction, increased oxidative stress and sperm DNA fragmentation. Importantly, these effects may occur not only in overt bacterial infection, but also in chronic or abacterial inflammatory states, where persistent immune activation and altered glandular function sustain a pro-oxidant seminal microenvironment [25,26,27,28].

Varicocele, the abnormal dilatation of the pampiniform venous plexus resulting from impaired venous drainage of the spermatic cord, affects approximately 15% of the general male population, but its prevalence rises to 35–40% among infertile men and up to 45–80% in those with secondary infertility [29]. Although many affected individuals remain fertile, varicocele has been associated with progressive testicular dysfunction, impaired spermatogenesis, sperm DNA damage, and chronic scrotal pain [30,31]. The mechanisms linking varicocele to testicular injury are complex and likely involve increased scrotal temperature, venous reflux, hypoxia, oxidative stress, and chronic inflammation. These mechanisms can increase ROS generation and reduce antioxidant defenses, leading to lipid peroxidation, mitochondrial dysfunction and sperm DNA damage. In this setting, oxidative stress is often considered a key mediator linking the vascular abnormality to impaired spermatogenesis and reduced sperm functional competence [17,32,33]. The marked clinical and biological heterogeneity suggests that molecular approaches may provide valuable insights into disease pathophysiology and help distinguish patients at risk of progressive reproductive impairment [34].

Systemic metabolic disorders also contribute to inflammatory and oxidative sperm injury [35]. Obesity, metabolic syndrome, and diabetes mellitus have reached epidemic proportions worldwide and are increasingly recognized as major contributors to male reproductive dysfunction [10,36]. These metabolic disorders impair spermatogenesis through a complex interplay of functional hypogonadism, insulin resistance, chronic low-grade inflammation, enhanced oxidative stress, adipokine dysregulation, and alterations of the testicular microenvironment [35,36].

Beyond conventional semen abnormalities, obesity and diabetes have been associated with biofunctional sperm defects, including increased DNA fragmentation, mitochondrial dysfunction, and altered fertilizing capacity, highlighting the multifactorial nature of metabolic male infertility [35,36].

In addition, metabolic dysfunction can alter mitochondrial activity and redox homeostasis, thereby affecting sperm motility and DNA integrity. In this context, seminal oxidative stress may reflect not only local reproductive tract injury, but also systemic immunometabolic imbalance [37,38]. Despite growing awareness, the molecular mechanisms linking metabolic disease to impaired male fertility remain partially understood, limiting both patient stratification and the development of targeted therapeutic strategies [36].

Environmental and lifestyle exposures represent an important source of male reproductive dysfunction, acting through overlapping endocrine, oxidative, inflammatory and epigenetic mechanisms. Endocrine-disrupting chemicals such as bisphenol A, phthalates, parabens and triclosan, together with pesticides, heavy metals and PFAS, have been associated with impaired semen quality, sperm DNA damage, mitochondrial dysfunction, altered steroidogenesis, disruption of the blood–testis barrier and defective sperm maturation [39,40,41,42]. These exposures rarely occur in isolation and may interact with smoking, alcohol intake, diet, heat exposure, air pollution, genetic susceptibility and pre-existing clinical conditions. Environmental exposures are particularly difficult to interpret because they often coexist and may interact with genetic susceptibility, lifestyle factors and pre-existing clinical conditions. Therefore, environmental injury should be considered an important contributor to heterogeneity in studies of sperm oxidative stress and proteomic remodeling [10,18,43].

Finally, advanced paternal age is increasingly recognized as an independent factor influencing male reproductive health. While conventional semen parameters show only modest age-related changes, growing evidence indicates that sperm quality progressively deteriorates at the molecular level [44,45]. In particular, aging is associated with increased sperm DNA fragmentation, oxidative damage, impaired chromatin integrity, mitochondrial dysfunction, and the progressive accumulation of de novo germline mutations arising from lifelong spermatogonial divisions [44,45]. These molecular alterations may differentially affect fertilization, embryo development, and offspring health [44,45]. The limited ability of conventional semen analysis to capture these age-related molecular defects highlights the need for more comprehensive approaches capable of characterizing the biological consequences of reproductive aging [45]. Unlike acute inflammatory conditions, aging-related injury is usually chronic and progressive, reflecting cumulative exposure to oxidative insults, reduced antioxidant efficiency and declining germ cell quality. This makes aging an important confounder in clinical and proteomic studies, particularly when comparing infertile cohorts with controls who are not age-matched.

### 2.2. Biochemical Readouts: ROS, Lipid Peroxidation, DNA Damage Markers, Cytokines, suPAR and sST2

The assessment of inflammatory and oxidative injury in male infertility requires the integration of complementary biochemical readouts. Direct or indirect measurement of ROS provides information on the oxidant burden within semen, whereas total antioxidant capacity reflects the ability of seminal plasma to buffer ROS production. However, both parameters are highly dynamic and influenced by abstinence time, leukocyte concentration, sample handling, assay platform and the relative contribution of spermatozoa versus seminal plasma. For this reason, isolated ROS or antioxidant measurements should be interpreted cautiously and, whenever possible, integrated with downstream markers of oxidative damage [4,17,43].

Lipid peroxidation is one of the most informative downstream readouts of sperm oxidative injury. Because sperm membranes are rich in polyunsaturated fatty acids, excessive ROS exposure can initiate peroxidative chain reactions that impair membrane fluidity, ion transport, mitochondrial activity and flagellar function. Lipid peroxidation products, including malondialdehyde, 4-hydroxynonenal and F2-isoprostanes, have therefore been proposed as markers of oxidative membrane damage and may correlate with reduced motility and fertilizing potential. Among these, F2-isoprostanes are increasingly considered robust indicators of lipid peroxidation, although their use in routine andrology remains limited by analytical and standardization issues [8,11,46].

Sperm DNA fragmentation represents a downstream marker of genomic injury and is frequently linked to oxidative stress. ROS can induce base oxidation, single- and double-strand breaks, chromatin crosslinking, and defective DNA packaging, particularly in spermatozoa with impaired chromatin compaction or incomplete protamination. Although sperm DNA fragmentation is not specific for oxidative damage, it provides clinically relevant information because it has been associated with reduced natural fertility, impaired assisted reproduction outcomes and increased risk of pregnancy loss in some settings. The combined assessment of oxidative stress and sperm DNA fragmentation may therefore offer a more complete picture than either parameter alone [26,47,48].

Inflammatory readouts include leukocytospermia, cytokines, chemokines and soluble immune mediators. Interleukins such as IL-6, IL-8, IL-1β and TNF-α have been widely investigated in semen and may reflect local immune activation, accessory gland inflammation or infection [49,50]. However, cytokine interpretation is complicated by biological variability, assay heterogeneity and the fact that low-grade inflammation may occur even below the conventional leukocytospermia threshold. Therefore, inflammatory biomarkers should ideally be interpreted in relation to clinical phenotype, microbiological findings, imaging evidence and oxidative stress markers rather than as standalone diagnostic tools [26,51,52].

In this framework, soluble urokinase plasminogen activator receptor, or suPAR, and soluble suppression of tumorigenicity 2, or sST2, are emerging as candidate biomarkers of seminal inflammation and inflammatory activity [24,53]. suPAR reflects activation of the urokinase plasminogen activator receptor system and has been studied as a systemic marker of immune activation and chronic inflammation, while sST2 acts as a soluble decoy receptor within the IL-33/ST2 axis and is implicated in inflammatory and tissue-remodeling responses [24]. Preliminary studies suggest that these markers may provide complementary information on inflammatory activation and MAGI-related phenotypes, particularly when integrated with oxidative stress assays and proteomic profiling [20,24,53]. However, the current evidence remains limited and largely derived from single-center or affiliated cohorts. Therefore, suPAR and sST2 should be regarded as exploratory or emerging biomarkers rather than validated clinical tools, and independent replication in larger, externally validated cohorts is required before their use can be recommended for routine diagnostic or prognostic stratification.

### 2.3. Major Sources of Heterogeneity and Misclassification

A major limitation in the study of inflammatory and oxidative injury in male infertility is clinical heterogeneity. Different conditions, such as MAGI, prostatitis, varicocele, obesity, diabetes, environmental exposure and aging, may converge on similar semen abnormalities while arising from distinct biological mechanisms. Conversely, patients with the same clinical diagnosis may display different inflammatory, redox and proteomic profiles depending on disease duration, anatomical site, bacterial status, leukocyte activation, antioxidant capacity and previous treatments. This explains why conventional categories such as “idiopathic infertility”, “leukocytospermia” or “oxidative stress-positive” often include biologically heterogeneous patients. Misclassification may also arise from the imperfect relationship between leukocytospermia and oxidative stress. Although leukocytes are an important source of ROS, oxidative stress may be present even in the absence of leukocyte counts above the conventional threshold. Conversely, leukocytospermia does not always translate into clinically relevant oxidative damage, particularly when seminal antioxidant capacity is preserved. Therefore, using leukocyte concentration alone as a surrogate for inflammatory–oxidative injury may underestimate some patients and overclassify others [26,51,54].

Assay-related heterogeneity is another critical issue. ROS measurement, total antioxidant capacity, lipid peroxidation assays, sperm DNA fragmentation tests and cytokine platforms differ substantially in analytical principles, pre-analytical requirements, sensitivity, specificity and clinical cut-offs. Variations in abstinence time, liquefaction, centrifugation, sperm purification, storage temperature, freeze–thaw cycles and contamination by leukocytes or immature germ cells may significantly influence results. These factors are particularly relevant for proteomic studies, where differences in sample preparation and data processing can affect protein recovery, quantification and pathway interpretation.

The cellular composition of semen is itself a source of misclassification. Whole semen, seminal plasma, purified spermatozoa and selected sperm subpopulations provide different biological information. Seminal plasma reflects accessory gland secretion and extracellular inflammatory mediators, whereas purified spermatozoa provide a more direct readout of sperm-intrinsic damage or remodeling. However, inadequate sperm purification may introduce proteins derived from leukocytes, epithelial cells, extracellular vesicles or residual plasma, potentially confounding the interpretation of sperm proteomic signatures. For this reason, rigorous sample processing and transparent reporting of purification methods are essential in studies addressing sperm proteomics under inflammatory and oxidative conditions.

Finally, temporal heterogeneity should not be underestimated. Inflammatory and oxidative injury may be acute, chronic, recurrent or partially compensated. Acute inflammation may produce a transient but intense oxidative burden, whereas chronic conditions may lead to adaptive or maladaptive remodeling of the seminal environment and sperm proteome. A single semen sample may therefore capture only one time point within a dynamic process. Longitudinal designs, paired pre/post-treatment analyses and integration of clinical, biochemical and proteomic data are likely to be more informative than cross-sectional comparisons alone. This is particularly relevant for translational studies aiming to identify biomarkers of treatment response or reproductive outcome.

Overall, the operational definition of inflammatory and oxidative sperm injury should combine clinical context, biochemical readouts and molecular profiling. Without this integrated framework, there is a risk of grouping together patients with different mechanisms of infertility, diluting biologically meaningful signals and generating inconsistent findings across studies. Proteomics offers a way to reduce this uncertainty, but only if applied to well-phenotyped cohorts with standardized sample handling, appropriate controls and careful interpretation of inflammatory and redox biomarkers.

## 3. Pre-Analytical Variables and Sperm Purity: The Main Confounders

In sperm proteomics, pre-analytical variables and sperm purity represent some of the most critical determinants of data reliability. Unlike many somatic cell models, human semen is a complex and heterogeneous biological fluid composed of spermatozoa, seminal plasma, leukocytes, epithelial cells, immature germ cells, extracellular vesicles and soluble proteins derived from different male accessory glands. Therefore, proteomic profiles obtained from semen-derived samples may reflect not only sperm-intrinsic molecular features, but also variable contributions from the seminal microenvironment and contaminating cell populations. This issue is particularly relevant in inflammatory and oxidative conditions, where leukocyte infiltration, epithelial shedding, altered glandular secretion and increased extracellular vesicle release may profoundly influence the detected proteome [4,55,56,57].

For this reason, sperm proteomic studies require rigorous control of the entire pre-analytical workflow, from semen collection to sperm isolation, washing, lysis and protein extraction. Variability in abstinence time, liquefaction, temperature, processing delay, centrifugation conditions and purification strategy can affect sperm motility, membrane integrity, oxidative status and protein recovery. In addition, incomplete removal of seminal plasma or non-sperm cells may generate misleading proteomic signatures, especially when highly abundant extracellular or immune-related proteins are interpreted as sperm-derived. Standardized reporting of semen handling and sperm purification procedures is therefore essential to improve reproducibility and comparability across studies [58,59,60,61].

This methodological issue becomes even more important when investigating inflammatory and oxidative injury. In these settings, the biological signal of interest may overlap with the main sources of contamination. Leukocytes, epithelial cells and spermatogenic cells are not merely passive contaminants; they are active producers of inflammatory mediators, oxidative enzymes and abundant proteins that may dominate the proteomic profile if not adequately removed. Similarly, seminal plasma carryovers may introduce accessory gland proteins, cytokines, complement components, proteases, antioxidant enzymes and extracellular vesicle-associated proteins. Without adequate purification and quality control, it may be difficult to distinguish true sperm proteomic remodeling from changes in the surrounding inflammatory seminal milieu.

### 3.1. Semen Collection and Handling: Abstinence, Time, Temperature and Liquefaction

Semen collection is the first and often underestimated source of variability in sperm proteomic studies. Abstinence duration can influence semen volume, sperm concentration, motility, oxidative stress, DNA fragmentation and the relative abundance of seminal plasma proteins. Although standard semen analysis usually recommends a defined abstinence interval, differences within this range may still affect both functional parameters and molecular readouts. Shorter abstinence may reduce sperm residence time in the epididymis and exposure to oxidative damage, whereas longer abstinence may increase sperm concentration but also enhance oxidative burden, DNA fragmentation and accumulation of senescent spermatozoa in some contexts [61,62,63,64].

Processing time after ejaculation is another crucial determinant. After collection, semen undergoes liquefaction, during which spermatozoa are exposed to a changing extracellular environment rich in proteases, vesicles, antioxidants, cytokines and accessory gland-derived proteins. Delayed processing may alter sperm motility, promote ROS accumulation, increase membrane damage and modify the balance between sperm-bound and soluble proteins. For proteomic studies, this means that time-to-processing should be standardized and explicitly reported, particularly when comparing fertile controls, infertile patients and inflammatory cohorts. The WHO manual emphasizes standardized semen examination procedures, including timing and handling conditions, precisely because these variables influence routine and advanced semen assessments [58,61].

Temperature control is equally relevant. Spermatozoa are sensitive to both cold shock and heat exposure, which can affect motility, membrane fluidity, mitochondrial function and oxidative status. Inconsistent temperature during transport, liquefaction or centrifugation may introduce technical variability that is particularly problematic for studies focused on redox imbalance and inflammation. Since oxidative injury can be experimentally amplified by inappropriate handling, poor temperature control may blur the distinction between disease-associated oxidative damage and artifact introduced during sample processing.

Liquefaction and sample homogenization also require careful standardization. Incomplete liquefaction, high viscosity or mucus-like material can impair accurate semen analysis and may affect sperm recovery during purification. This is especially relevant in prostatitis, MAGI and accessory gland dysfunction, where altered seminal coagulation/liquefaction dynamics may reflect inflammatory changes in glandular secretion. From a proteomic perspective, abnormal viscosity may increase seminal plasma carryover, reduce washing efficiency and favor retention of extracellular proteins on the sperm surface. Thus, liquefaction status should not be regarded only as a routine macroscopic parameter, but also as a potential determinant of sperm proteome purity.

Overall, semen collection and handling variables should be considered part of the experimental design rather than simple technical details. In studies addressing inflammatory and oxidative sperm injury, harmonization of abstinence interval, collection method, liquefaction time, processing delay, temperature and storage conditions is necessary to avoid introducing pre-analytical noise into downstream proteomic analyses.

### 3.2. Sperm Isolation Strategies and Contamination: Leukocytes, Epithelial and Spermatogenic Cells and Seminal Plasma Carryover

**Figure 1:**
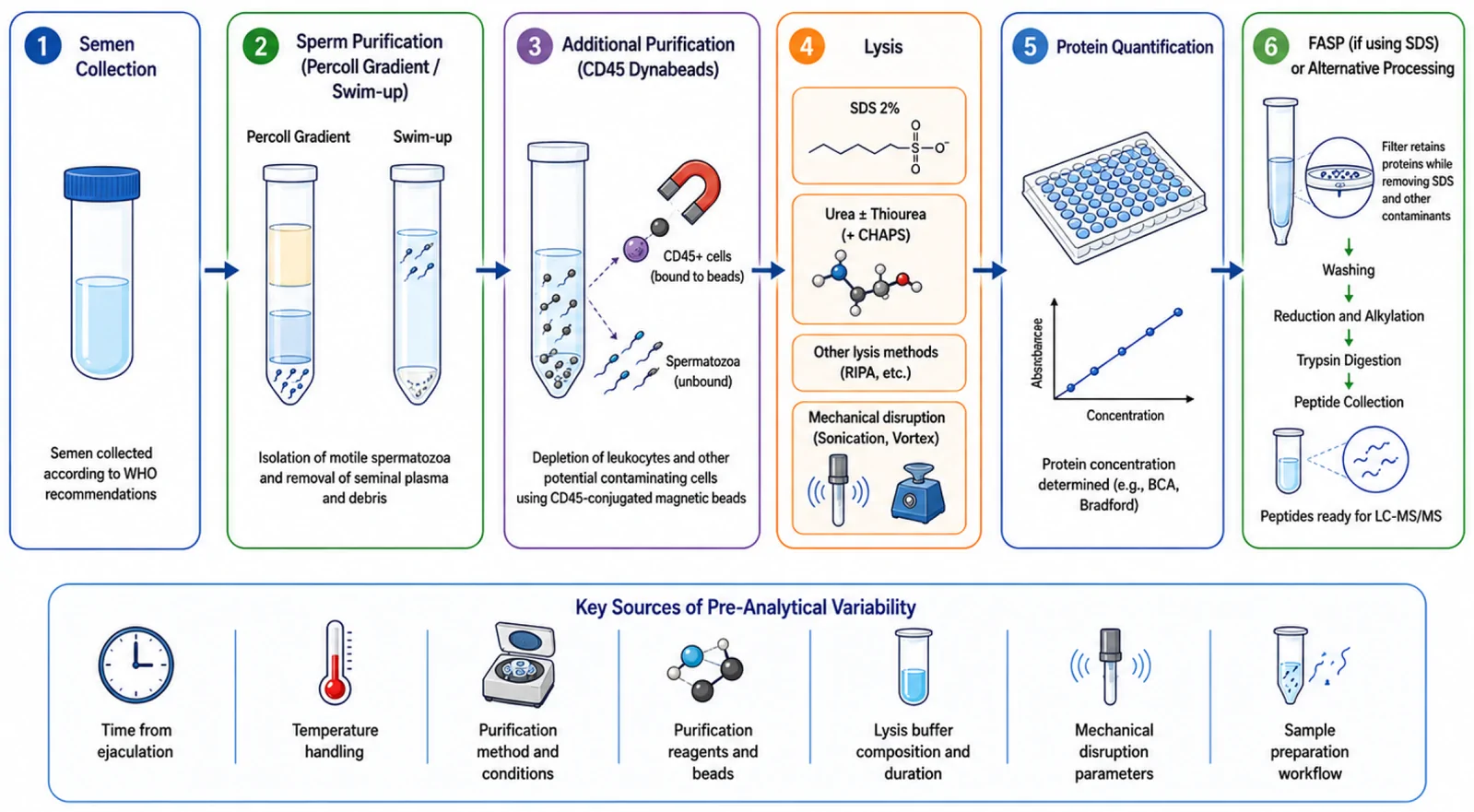
Pre-analytical workflow for sperm proteomic analysis. Schematic representation of the main steps preceding mass spectrometry-based sperm proteomics, including semen collection, sperm isolation by density-gradient centrifugation or swim-up, optional additional purification using immunomagnetic beads, protein extraction using different lysis strategies, protein quantification, and downstream sample processing. When SDS-based lysis is employed, detergent removal may be achieved by Filter-Aided Sample Preparation (FASP) before enzymatic digestion. Each step represents a potential source of pre-analytical variability that may affect protein recovery, sample purity, and the resulting sperm proteomic profile.

The choice of sperm isolation strategy has a major impact on the proteomic profile obtained from human spermatozoa. Common approaches include simple washing, swim-up and density gradient centrifugation, each of which selects partially different sperm populations and removes contaminants with different efficiency. Simple washing may preserve a broader sperm population, but it is less effective in eliminating seminal plasma proteins, leukocytes, epithelial cells and immature germ cells. Swim-up enriches for highly motile spermatozoa, whereas density gradient centrifugation allows recovery of a cleaner and often more functionally competent sperm fraction. Both methods help in eliminating non-nemaspermic cells such as epithelial and spermatogenic cells, but both may introduce selection bias, because the isolated spermatozoa may not fully represent the whole ejaculate [55,56,65,66]. Contamination by leukocytes is one of the most critical confounders in studies of inflammatory sperm injury. Leukocytes contain abundant cytoskeletal, metabolic, immune and oxidative enzymes that can be detected by mass spectrometry and mistakenly assigned to spermatozoa if sample purity is not ensured. This is particularly problematic because leukocyte-derived proteins may overlap with pathways of interest, including inflammation, oxidative stress, proteolysis, complement activation and innate immune response. Therefore, when proteomic studies report enrichment of immune or inflammatory pathways in sperm preparations, it is essential to verify whether these signals derive from genuine sperm-associated remodeling or from residual leukocyte contamination [51,56,67]. Seminal plasma carryover is another major issue. Seminal plasma is rich in highly abundant proteins, including prostate- and seminal vesicle-derived proteins, enzymes, binding proteins, cytokines, antioxidants, immunoglobulins, complement proteins and extracellular vesicle cargo. Even small residual amounts may influence proteomic results, particularly because seminal plasma proteins can adhere to the sperm surface or be retained after inadequate washing. This is relevant in inflammation, where the seminal plasma proteome itself is remodeled and may contain increased inflammatory mediators, acute-phase proteins, proteases and oxidative stress-related proteins. Therefore, sperm washing steps and assessment of seminal plasma depletion should be explicitly reported. At the same time, complete separation between sperm-intrinsic and sperm-associated proteins is biologically complex. During ejaculation and post-ejaculatory maturation, spermatozoa interact with seminal plasma proteins and extracellular vesicles, including prostasomes and epididymosomes. Some proteins detected in sperm preparations may therefore represent true sperm-associated surface or vesicle-transferred components rather than simple seminal plasma carryover. This distinction is important because extracellular vesicle-mediated transfer is increasingly recognized as part of sperm maturation and functional regulation. However, for proteomic interpretation, authors should clearly distinguish between “sperm-intrinsic”, “sperm-associated” and “possible contaminant-derived” proteins whenever possible [68,69,70]. In this context, the methodological work of Rafael Oliva and colleagues has been particularly influential in defining how sperm proteomic studies should address sample purity and biological interpretability. Their protocols emphasize that, before protein extraction and mass spectrometry analysis, spermatozoa should be carefully separated from seminal plasma and non-sperm cells, generally using density gradient-based purification followed by repeated washing and microscopic quality assessment. In methodological descriptions from this group, sperm purification is not treated as a minor preparatory step, but as a central prerequisite to ensure that the detected proteome can be reliably attributed to spermatozoa rather than to leukocytes, epithelial cells, immature germ cells or residual seminal plasma proteins [55,66,71]. It has been reported that residual leukocytes can be depleted using a combination of density-gradient purification and anti-CD45-conjugated Dynabeads, thereby improving sperm sample purity before downstream proteomic analysis [71]. Following magnetic depletion, sample purity was carefully verified, strengthening the attribution of the identified proteins to spermatozoa rather than contaminating leukocytes. Beyond technical purification, the work of Oliva’s group has also shaped the broader interpretation of sperm proteomics. Their studies and reviews highlighted that mature spermatozoa are particularly suitable for proteomic investigation because they are accessible, can be purified, and are largely transcriptionally and translationally silent; consequently, their protein composition represents a relatively stable molecular readout of spermatogenesis, epididymal maturation, capacitation-related remodeling and pathological injury [59,70,72,73]. In this view, sperm proteomics is not only a biomarker discovery strategy, but also a functional approach to identify the molecular networks underlying motility, chromatin organization, energy metabolism, membrane remodeling and fertilization competence. In addition to cellular purity, functional quality assessment of the purified sperm fraction should be considered an important pre-analytical component of proteomic studies. Evaluation of sperm viability, apoptosis-like markers, mitochondrial membrane potential, ROS production and DNA integrity, including by flow cytometry-based approaches, can provide essential contextual information for interpreting proteomic alterations under inflammatory and oxidative conditions. Integrating these functional readouts with sperm proteomics may help distinguish intrinsic sperm damage from selective enrichment of specific sperm subpopulations, thereby improving the translational relevance of proteomic findings. Following sperm purification, protein extraction represents an additional critical source of pre-analytical variability. Mature spermatozoa are highly specialized cells characterized by a compact architecture, extensive cytoskeletal structures, specialized membranes, and highly condensed chromatin, making efficient and reproducible protein solubilization particularly challenging [59]. Accordingly, markedly different extraction strategies have been employed across sperm proteomic studies. Strong detergent-based lysis, particularly using sodium dodecyl sulfate (SDS), provides efficient solubilization of a broad range of sperm proteins, including membrane-associated and poorly soluble components. When coupled with detergent-removal workflows such as filter-aided sample preparation (FASP), SDS-based extraction can be effectively integrated with bottom-up LC-MS/MS, combining efficient sperm disruption with subsequent protease-compatible sample processing [71,74]. Conversely, several studies have employed chaotropic extraction buffers containing high concentrations of urea, frequently combined with thiourea and the zwitterionic detergent CHAPS. For example, urea/thiourea-based extraction has been used in proteomic studies of human sperm selected by swim-up [75], whereas 8 M urea-based buffers have also been applied to the characterization of specific sperm subcellular compartments, including the flagellar proteome [76]. Urea/thiourea/CHAPS formulations have been particularly common in workflows involving two-dimensional electrophoresis and MALDI-TOF/TOF analysis, although related chaotropic formulations have subsequently been adapted to MS-based quantitative proteomic workflows [77]. Mechanical disruption, particularly sonication, is frequently combined with chemical lysis to improve protein extraction from the highly compact sperm structure. Other approaches include detergent-based extraction buffers and sequential fractionation protocols designed to recover proteins with different solubility properties or to investigate specific subcellular compartments, such as the sperm tail or chromatin-associated proteome [71,76,78]. Importantly, these extraction procedures should not be considered analytically equivalent. Different extraction strategies may preferentially recover distinct subsets of the sperm proteome, particularly with respect to membrane-associated, cytoskeletal, nuclear, and poorly soluble proteins, thereby contributing to inter-study variability in protein identification and pathway representation [59]. This methodological issue may be particularly relevant when investigating inflammatory and oxidative injury. Incomplete extraction of poorly soluble structural proteins may lead to under-representation of flagellar and cytoskeletal components, whereas inefficient membrane solubilization may reduce the detection of membrane-associated proteins and ion channels involved in sperm motility and fertilization. Consequently, apparent differences in functional categories across sperm proteomic studies may partly reflect sample-processing procedures in addition to genuine biological variability. Emerging sample-preparation technologies, including suspension trapping (S-Trap) and single-pot solid-phase-enhanced sample preparation (SP3), offer potential advantages by enabling efficient detergent removal, reduced sample handling, and improved protein recovery, particularly from limited biological material [79,80,81]. However, their systematic application and direct benchmarking in human sperm proteomics remain less established than conventional chaotropic extraction or SDS-based workflows coupled with detergent-removal strategies. Standardized reporting of lysis buffer composition, mechanical disruption, protein-cleanup strategy, and digestion workflow is therefore essential to improve reproducibility and cross-study comparability. The choice of lysis buffer also influences the subsequent sample-processing workflow. In particular, when strong ionic detergents such as SDS are used for efficient sperm protein solubilization, they must be removed before enzymatic digestion and LC-MS/MS analysis because of their incompatibility with protease activity and mass spectrometry. FASP approach provides an effective solution by retaining proteins on a molecular-weight cut-off filter while allowing SDS and other low-molecular-weight contaminants to be removed through repeated washing, followed by on-filter reduction, alkylation, and enzymatic digestion [74]. Conversely, FASP is not necessarily required when detergent-free or chaotropic extraction protocols are used. For example, urea-based lysates can be processed by reducing the chaotrope concentration to levels compatible with protease activity and subsequently performing in-solution digestion, whereas gel-based urea/thiourea/CHAPS workflows may rely on in-gel digestion. Thus, the downstream protein-processing strategy should be selected according to the lysis chemistry employed, and both steps should be considered together when comparing proteomic workflows across studies. Overall, the main pre-analytical steps preceding sperm proteomic analysis—from semen collection and sperm isolation to additional purification, protein extraction, quantification, and downstream sample processing—are summarized in Figure 1, highlighting the major methodological alternatives that may influence the resulting proteomic profile. In summary, sperm isolation is not a neutral technical step but a biological filter that shapes the proteomic result. In the context of inflammatory and oxidative injury, this filter can either improve interpretability by removing confounding material or introduce bias by selecting specific sperm subpopulations. For this reason, studies should report not only the proteomic workflow, but also cellular purity, isolation rationale, potential contamination by non-sperm cells and carryover of seminal plasma components. Only under these conditions can sperm proteomics reliably distinguish intrinsic sperm damage from alterations of the inflammatory seminal environment.

## 4. Proteomics Workflows Applied to Spermatozoa

### 4.1. What Proteomics Adds Beyond Conventional Semen Analysis and Single Biomarkers

Despite its central role in the clinical evaluation of male infertility, conventional semen analysis remains an imperfect predictor of male reproductive potential. Proteomic studies have shown that clinically relevant conditions may be associated with sperm protein remodeling even when conventional semen parameters provide only partial biological information, supporting the value of proteomics as a complementary molecular readout of sperm function [82]. Standard parameters—including sperm concentration, motility, morphology, and vitality, as recommended by the World Health Organization (WHO)—provide valuable phenotypic information but offer only limited insight into the molecular mechanisms underlying sperm dysfunction. Indeed, a considerable proportion of infertile men present with normal semen parameters, a condition commonly referred to as idiopathic or unexplained male infertility, underscoring the limited diagnostic accuracy of routine semen analysis alone [83,84]. Several complementary diagnostic assays have been introduced to improve the evaluation of sperm quality, including measurements of reactive oxygen species (ROS), total antioxidant capacity, sperm DNA fragmentation, chromatin integrity, apoptosis markers, and genetic abnormalities. Although these tests provide important functional information, each assesses only a specific aspect of sperm biology and therefore cannot fully capture the complex molecular networks governing male fertility [85].

Proteomic profiling addresses these limitations by providing a comprehensive characterization of the protein landscape of spermatozoa and seminal plasma. Because proteins are the primary functional effectors of cellular processes, proteomics offers a direct molecular snapshot of the biological pathways regulating spermatogenesis, epididymal maturation, sperm motility, capacitation, acrosome reaction, sperm–oocyte interaction, and early embryonic development [86,87]. In contrast to conventional single-biomarker approaches, which investigate individual molecules in isolation, proteomic analyses simultaneously quantify thousands of proteins and their interactions, enabling the identification of coordinated molecular signatures associated with fertility or pathological conditions. This systems-level perspective has revealed distinct proteomic profiles associated with oxidative stress, inflammation, asthenozoospermia, teratozoospermia, varicocele, and unexplained male infertility, thereby uncovering molecular alterations that remain undetectable by routine semen analysis [86,88]. Furthermore, continuous advances in high-resolution mass spectrometry, quantitative proteomic workflows, and bioinformatic analyses have considerably expanded the capacity to identify clinically relevant protein biomarkers with potential diagnostic, prognostic, and therapeutic value.

Beyond biomarker discovery, proteomics has substantially advanced our understanding of the molecular mechanisms underlying male infertility by linking alterations in protein abundance to specific biological pathways and cellular functions. The integration of proteomic profiles with functional assays and clinical phenotypes is therefore paving the way toward precision medicine strategies, enabling improved patient stratification and the development of individualized diagnostic and therapeutic approaches [84,87]. Overall, proteomics complements conventional semen analysis by shifting the assessment of male infertility from a predominantly descriptive evaluation of sperm quality to a mechanistic understanding of the molecular pathways that govern sperm function and reproductive competence. This transition represents a fundamental step toward the implementation of molecular diagnostics and personalized reproductive medicine.

### 4.2. Bottom-Up LC–MS/MS (DDA vs. DIA) and Quantitative Strategies (Label-Free vs. Isobaric)

**Figure 2:**
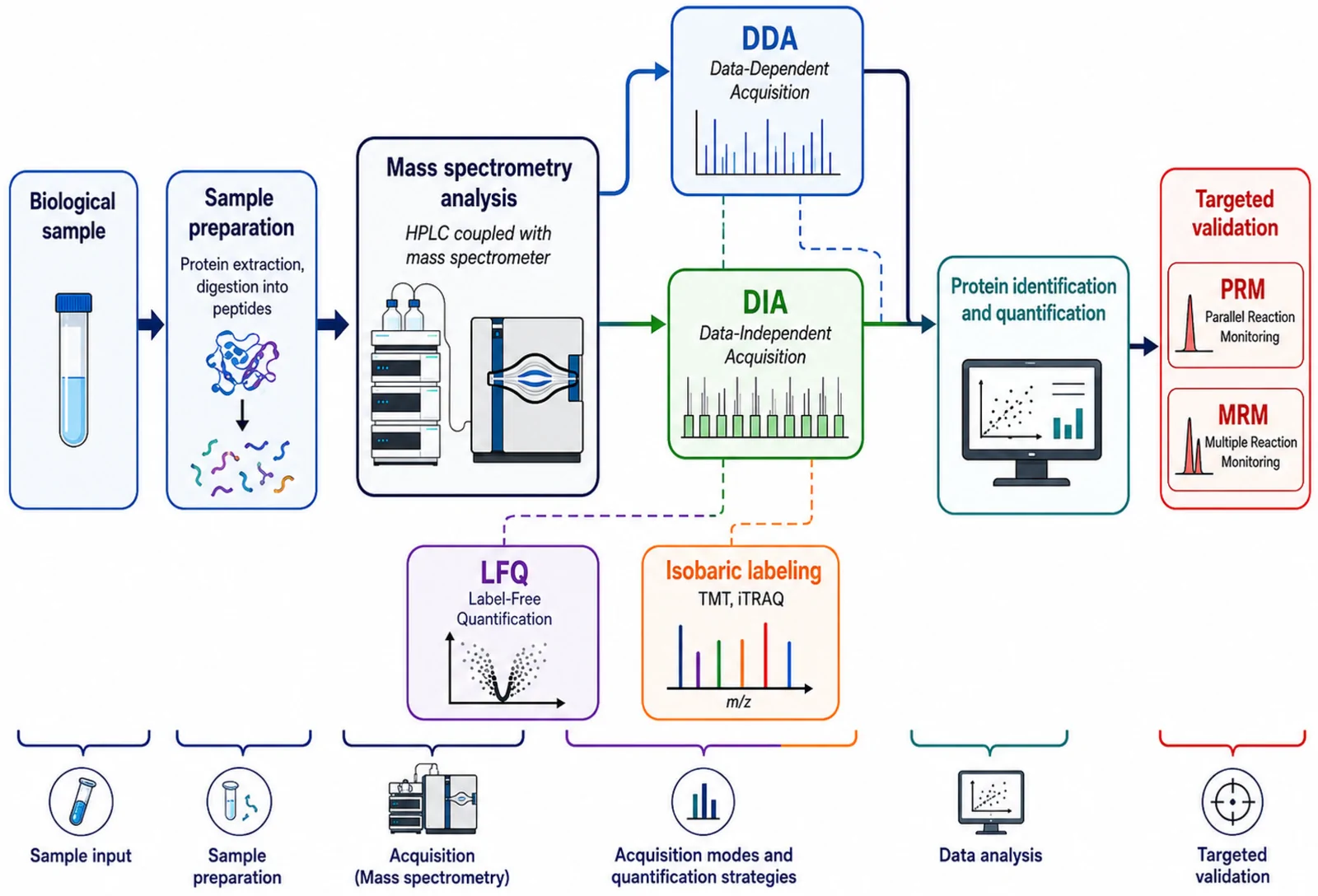
Overview of the bottom-up proteomics workflow applied to spermatozoa and seminal plasma. Following biological sample preparation and enzymatic protein digestion, peptides are analyzed by LC–MS/MS using data-dependent acquisition (DDA) or data-independent acquisition (DIA). Protein quantification can be performed through label-free quantification (LFQ) or isobaric labeling approaches, such as tandem mass tags (TMT) and isobaric tags for relative and absolute quantification (iTRAQ). Candidate biomarkers identified during discovery proteomics are subsequently assessed through targeted mass spectrometry, including parallel reaction monitoring (PRM) and multiple reaction monitoring (MRM).

The rapid evolution of mass spectrometry (MS)-based proteomics has revolutionized the characterization of the molecular landscape of spermatozoa and seminal plasma. Among the available analytical platforms, bottom-up liquid chromatography coupled with tandem mass spectrometry (LC–MS/MS) has become the gold standard for large-scale protein identification and quantification in reproductive research. In this workflow, proteins are enzymatically digested into peptides prior to chromatographic separation and mass spectrometric analysis, enabling comprehensive and highly sensitive profiling of complex biological samples. Owing to its broad proteome coverage, analytical robustness, and ability to identify thousands of proteins from limited biological material, bottom-up LC–MS/MS has become the cornerstone of contemporary sperm proteomics [86,87]. The overall workflow of bottom-up proteomics, from biological sample preparation to mass spectrometric acquisition, quantitative analysis, and targeted biomarker validation, is summarized in Figure 2.

Data-dependent acquisition (DDA) has long been the predominant approach in proteomic studies of male infertility. In DDA workflows, the mass spectrometer performs an initial survey scan (MS1) and automatically selects the most abundant precursor ions for fragmentation and peptide sequencing. This approach has been instrumental in generating comprehensive proteomic maps of spermatozoa and seminal plasma and has facilitated the identification of proteins associated with asthenozoospermia, varicocele, oxidative stress, and idiopathic male infertility [86,88]. However, because precursor selection is inherently stochastic, DDA often suffers from limited reproducibility across independent analyses and a high frequency of missing values, particularly for proteins expressed at low abundance. These limitations become increasingly evident in large clinical studies, where reproducibility and quantitative consistency are essential.

To address these challenges, data-independent acquisition (DIA) has emerged as a powerful alternative for quantitative proteomics. Rather than selecting only the most intense precursor ions, DIA systematically fragments all peptide ions within predefined mass-to-charge (*m*/*z*) windows, thereby generating a more comprehensive and unbiased representation of the proteome. This acquisition strategy substantially improves reproducibility, quantitative precision, and proteome coverage while enhancing the detection of low-abundance proteins. Consequently, DIA has become particularly attractive for biomarker discovery and clinical proteomics, where accurate comparison of large patient cohorts is required. Recent studies indicate that DIA-based workflows outperform conventional DDA in both analytical depth and quantitative robustness, making them increasingly suitable for translational applications in male infertility research [89].

More recently, library-free DIA strategies have further simplified DIA-based proteomic workflows by reducing the need for separately acquired DDA spectral libraries. Approaches such as direct DIA have increased the flexibility of DIA analysis and reduced its dependence on experimentally generated libraries, highlighting the rapid evolution of DIA-based proteomic workflows [89]. In parallel with advances in acquisition methods, significant progress has also been achieved in quantitative proteomic strategies. These can be broadly classified into label-free and isobaric labeling approaches, each offering distinct advantages depending on the experimental design. Label-free quantification (LFQ) estimates protein abundance directly from peptide signal intensities or spectral counts without requiring chemical or metabolic labeling. Because of its relatively simple experimental workflow, reduced costs, and virtually unlimited sample capacity, LFQ has become one of the most widely adopted quantitative approaches in reproductive proteomics. Its scalability makes it particularly suitable for large clinical cohorts frequently encountered in male infertility studies [83,84]. Nevertheless, quantitative accuracy depends on careful normalization and instrument stability, as measurements acquired in separate LC–MS/MS runs remain susceptible to technical variability.

Alternatively, isobaric labeling strategies, including tandem mass tags (TMT) and isobaric tags for relative and absolute quantification (iTRAQ), enable multiplexed quantitative analysis by chemically labeling peptides from different biological samples with isobaric reagents prior to sample pooling. During tandem mass spectrometry, peptide fragmentation releases reporter ions whose relative intensities reflect protein abundance across the multiplexed samples. By allowing simultaneous analysis of multiple biological conditions within a single LC–MS/MS experiment, isobaric labeling substantially reduces technical variability, improves experimental throughput, and enhances quantitative precision across study groups [85,86]. However, these approaches require specialized reagents and remain susceptible to ratio compression caused by co-isolation and co-fragmentation of interfering peptides, which may reduce quantitative accuracy for proteins exhibiting subtle expression differences.

The combination of advanced acquisition strategies with increasingly sophisticated quantitative methodologies has transformed reproductive proteomics from descriptive protein cataloguing into a robust systems biology approach capable of defining disease-associated molecular networks. While DDA combined with label-free quantification remains widely employed in exploratory investigations, the progressive adoption of DIA workflows and multiplexed isobaric labeling is expected to improve reproducibility, increase analytical depth, and accelerate the identification of clinically relevant biomarkers. As these technologies continue to mature, they are likely to play an increasingly important role in the translation of sperm proteomics into routine clinical practice.

### 4.3. Targeted Validation (PRM/SRM) and Orthogonal Confirmation (IF/WB)

Although discovery-based proteomic approaches have considerably expanded our understanding of the molecular alterations underlying male infertility, the identification of differentially expressed proteins alone is insufficient for clinical translation. Candidate biomarkers identified through large-scale LC–MS/MS analyses must be rigorously validated in independent patient cohorts to demonstrate their reproducibility, biological relevance, and potential diagnostic or prognostic value. Consequently, targeted mass spectrometry and orthogonal validation techniques represent essential steps in the proteomic pipeline, bridging the gap between biomarker discovery and clinical implementation [84,85].

Among targeted proteomic approaches, selected reaction monitoring (SRM)—also referred to as multiple reaction monitoring (MRM)—is considered the reference method for the quantitative verification of candidate biomarkers. Typically performed on triple quadrupole mass spectrometers, SRM selectively monitors predefined precursor-to-fragment ion transitions corresponding to proteotypic peptides. This highly targeted strategy provides excellent analytical sensitivity, quantitative accuracy, and inter-run reproducibility, making it particularly suitable for validating proteins identified during discovery-based studies. In reproductive proteomics, SRM has been successfully applied to confirm alterations in proteins involved in sperm motility, mitochondrial metabolism, oxidative stress responses, and fertilization competence, thereby strengthening the evidence supporting their potential clinical relevance [86].

More recently, parallel reaction monitoring (PRM) has emerged as a powerful alternative to SRM. Unlike SRM, which monitors only a limited number of predefined fragment ions, PRM acquires the complete fragmentation spectrum of a selected precursor ion using high-resolution, high-mass-accuracy instruments such as Orbitrap mass spectrometers. The simultaneous detection of all product ions increases analytical specificity, simplifies assay development, and reduces interference from co-eluting peptides, thereby improving confidence in peptide identification and quantification [84,90]. Owing to these advantages, PRM is increasingly adopted for the validation of low-abundance proteins and candidate biomarkers identified in spermatozoa and seminal plasma, particularly when high analytical accuracy is required.

While targeted mass spectrometry provides robust quantitative confirmation of protein abundance, orthogonal validation techniques supply complementary biological evidence that strengthens the interpretation of proteomic findings. Among these, Western blotting (WB) remains the most widely used method for confirming differential protein expression identified by LC–MS/MS analyses. Using highly specific antibodies, Western blotting independently verifies changes in protein abundance across experimental groups and has therefore become a standard validation tool in studies investigating oxidative stress, varicocele, asthenozoospermia, and other forms of male infertility [85,86].

In addition to quantitative confirmation, immunofluorescence (IF) microscopy provides valuable spatial information by defining the subcellular localization of candidate proteins within distinct sperm compartments, including the acrosome, nucleus, equatorial segment, midpiece, and flagellum. This information is particularly relevant because sperm function depends not only on protein abundance but also on the precise localization of proteins involved in capacitation, hyperactivation, acrosome reaction, and sperm–oocyte interaction. Consequently, immunofluorescence complements quantitative proteomic analyses by linking protein expression to its functional context, thereby providing mechanistic insight into the biological significance of proteomic alterations [91].

Collectively, targeted mass spectrometry and orthogonal validation approaches constitute indispensable components of modern reproductive proteomics. A typical validation workflow begins with the identification of differentially expressed proteins through discovery-based LC–MS/MS analyses, followed by quantitative verification using SRM or PRM and independent confirmation through Western blotting and immunofluorescence. This multi-layered strategy substantially increases confidence in candidate biomarkers, improves reproducibility across independent studies, and facilitates the translation of proteomic discoveries into clinically applicable diagnostic tools.

Beyond biomarker validation, these complementary methodologies also contribute to defining the molecular pathways that underpin male infertility. By integrating quantitative proteomics with targeted and functional validation, it is possible to identify reproducible protein networks associated with oxidative stress, inflammation, mitochondrial dysfunction, impaired motility, defective capacitation, and reduced fertilization competence. These interconnected molecular signatures provide the conceptual framework for the following chapter, which examines the core proteomic pathways involved in oxidative and inflammatory injury. The main proteomic workflows discussed in this section, including discovery approaches, quantitative strategies, targeted validation and orthogonal confirmation methods, are summarized in Table 1.

**Table 1:** Overview of the principal proteomic workflows currently employed in male infertility research. Discovery proteomics is primarily performed using bottom-up LC–MS/MS with either data-dependent acquisition (DDA) or data-independent acquisition (DIA). Quantitative analyses can be achieved through label-free quantification (LFQ) or isobaric labeling approaches (TMT/iTRAQ), whereas candidate biomarkers are validated using targeted mass spectrometry (SRM/MRM or PRM) and orthogonal techniques such as Western blotting (WB) and immunofluorescence (IF). Together, these complementary approaches provide a robust framework for biomarker discovery, validation, and clinical translation.

Workflow Step	Main Techniques	Strengths	Limitations	Typical Applications in Male Infertility
**Protein identification (Discovery proteomics)**	Bottom-up LC–MS/MS (DDA)	High proteome coverage; well-established workflows; extensive spectral libraries	Stochastic precursor selection; missing values; limited reproducibility across runs	Exploratory studies; protein identification; comparative proteomics
**Protein identification (Discovery proteomics)**	Bottom-up LC–MS/MS (DIA)	Comprehensive peptide acquisition; improved reproducibility; enhanced detection of low-abundance proteins	More complex data processing; requires dedicated computational tools	Large cohort studies; biomarker discovery; clinical proteomics
**Quantitative proteomics**	Label-Free Quantification (LFQ)	Cost-effective; simple workflow; unlimited number of samples; suitable for clinical cohorts	Sensitive to run-to-run variability; requires robust normalization	Differential protein expression analysis in large patient cohorts
**Quantitative proteomics**	Isobaric labeling (TMT, iTRAQ)	Multiplex analysis; reduced technical variability; high quantitative precision	Higher cost; ratio compression due to co-isolation interference	Comparative studies involving multiple experimental groups
**Targeted validation**	Selected/Multiple Reaction Monitoring (SRM/MRM)	High sensitivity; excellent quantitative accuracy; robust reproducibility	Limited multiplexing capability; assay development required	Verification of candidate protein biomarkers
**Targeted validation**	Parallel Reaction Monitoring (PRM)	High specificity; simplified assay development; accurate quantification using high-resolution MS	Requires Orbitrap or equivalent high-resolution instrumentation	Validation of low-abundance proteins and clinical biomarker candidates
**Orthogonal validation**	Western blotting (WB)	Independent confirmation of protein abundance; widely available	Antibody-dependent; low throughput	Validation of differential protein expression identified by LC–MS/MS
**Orthogonal validation**	Immunofluorescence (IF)	Provides spatial localization of proteins within sperm compartments; supports functional interpretation	Qualitative or semi-quantitative; dependent on antibody quality	Confirmation of protein localization during capacitation, acrosome reaction, and sperm–oocyte interaction

## 5. Core Proteomic Signatures of Oxidative and Inflammatory Injury in Male Infertility

**Figure 3:**
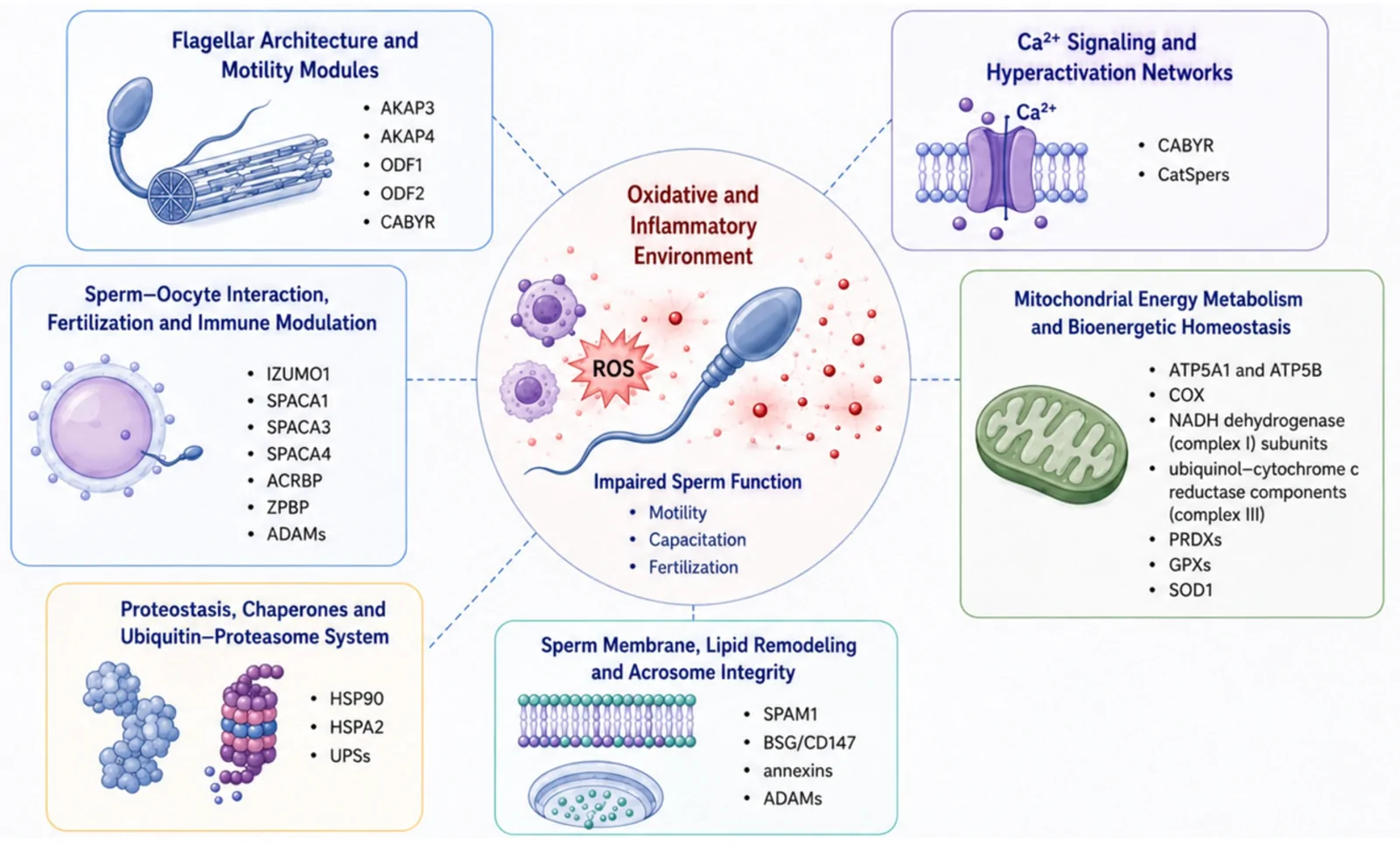
Core proteomic signatures of oxidative and inflammatory injury in male infertility. Schematic representation of the major interconnected molecular pathways affected by oxidative and inflammatory stress in spermatozoa, including flagellar architecture and motility, Ca^2+^ signaling and hyperactivation, mitochondrial bioenergetics and redox homeostasis, membrane remodeling and capacitation, proteostasis and stress-response pathways, and sperm–oocyte interaction. Representative proteins reported as dysregulated within each pathway are shown. Collectively, these alterations converge on impaired sperm motility, capacitation, and fertilization competence.

Over the past decade, proteomic technologies have considerably advanced our understanding of oxidative stress and inflammation mechanisms contributing to male infertility by enabling the large-scale characterization of molecular alterations occurring in both spermatozoa and seminal plasma. Rather than identifying isolated protein changes, contemporary proteomic studies have revealed coordinated alterations affecting interconnected biological pathways involved in antioxidant defense, energy metabolism, calcium signaling, cytoskeletal organization, membrane remodeling, protein quality control, and sperm–oocyte interaction. These molecular perturbations ultimately converge on the functional hallmarks of male infertility, including impaired motility, defective capacitation, compromised fertilization competence, and reduced reproductive outcomes. Collectively, these findings support the concept that oxidative stress is not merely a consequence of impaired fertility but represents a primary driver of sperm dysfunction capable of extensively remodeling the sperm proteome. The following sections examine the principal molecular pathways identified by reproductive proteomics, highlighting how alterations in protein networks governing motility, calcium signaling, mitochondrial metabolism, membrane dynamics, proteostasis, and sperm–oocyte interaction contribute to male infertility. These core proteomic signatures and their major dysregulated proteins are summarized in Figure 3, illustrating the interconnected molecular pathways through which oxidative and inflammatory stress converge on impaired sperm function. To facilitate interpretation of the evidence supporting these candidates, Table 2 summarizes the main proteins discussed across the mentioned functional modules, indicating the reported direction or type of alteration, the associated infertility phenotype, and the current level of validation. This distinction is clinically relevant because most sperm proteomic candidates remain at the discovery or early-validation stage and require targeted confirmation before translation into routine diagnostic assays.

### 5.1. Flagellar Architecture and Motility Modules

Progressive sperm motility is one of the most energy-demanding and tightly regulated functions of the male gamete and represents one of the earliest cellular processes affected by oxidative and inflammatory stress. Since efficient motility is essential for sperm transport through the female reproductive tract and successful fertilization, alterations in the molecular machinery controlling flagellar movement are consistently observed in infertile men, particularly in patients with asthenozoospermia. The sperm flagellum is a highly specialized organelle composed of the axoneme, outer dense fibers (ODFs), fibrous sheath, and associated regulatory complexes that coordinate ATP utilization, signal transduction, and mechanical force generation [92,93]. Proteomic analyses have demonstrated that oxidative stress profoundly affects both structural proteins and signaling molecules responsible for maintaining flagellar integrity and coordinated movement [86].

Among the proteins most consistently dysregulated are the A-kinase anchoring proteins-3 (AKAP3) and A-kinase anchoring proteins-4 (AKAP4), two major components of the fibrous sheath that organize signaling complexes regulating protein kinase A (PKA)-dependent pathways, glycolytic enzymes, and cytoskeletal organization. Reduced expression of AKAP4, in particular, has repeatedly been associated with decreased progressive motility, impaired fertilization potential, and poor outcomes following assisted reproductive technologies. Likewise, alterations in AKAP3 have been linked to defective capacitation and reduced flagellar function.

Proteomic studies have also identified significant changes in proteins responsible for maintaining the structural stability of the flagellum, including outer dense fiber proteins-1 and -2 (ODF1 and ODF2), dynein heavy chains, tektins, and the calcium-binding protein CABYR. In particular, decreased ODF2 protein levels have been reported as a marker of progressive detrimental effects in chronic inflammation, suggesting an intriguing link between sustained inflammatory injury and sperm functional impairment [24]. These proteins contribute to axonemal organization, force generation, and the regulation of flagellar beating. Their differential expression in asthenozoospermic patients indicates that oxidative stress affects not only sperm metabolism but also the structural architecture required for efficient motility [86,94].

The detrimental effects of reactive oxygen species extend beyond structural damage. Oxidative modifications of cytoskeletal proteins, together with impaired ATP production and disruption of phosphorylation-dependent signaling pathways, further compromise flagellar function. Since motility depends on the coordinated integration of energy metabolism, calcium signaling, and cytoskeletal dynamics, oxidative stress simultaneously disrupts multiple regulatory networks that collectively determine sperm movement.

Importantly, recent proteomic evidence indicates that abnormalities in flagellar proteins rarely occur as isolated events. Instead, they are closely associated with concurrent alterations in mitochondrial bioenergetics, antioxidant defense systems, and calcium-dependent signaling pathways, emphasizing the extensive molecular crosstalk underlying sperm dysfunction. These observations reinforce the concept that impaired motility represents the phenotypic consequence of widespread remodeling of interconnected protein networks rather than the dysregulation of individual proteins.

Consequently, the flagellar proteome has emerged as one of the most robust sources of candidate biomarkers for oxidative stress-mediated male infertility. Proteins such as AKAP4, AKAP3, CABYR, and ODF2 are consistently identified across independent proteomic studies and represent promising molecular indicators of sperm functional competence and fertilization potential.

### 5.2. Ca^2+^ Signaling and Hyperactivation Networks

Calcium signaling is a central regulator of sperm physiology, orchestrating a wide range of processes required for successful fertilization, including capacitation, hyperactivation, chemotaxis, acrosome reaction, and sperm–oocyte fusion [95,96]. The acquisition of hyperactivated motility, characterized by asymmetrical high-amplitude flagellar beating, depends on a tightly controlled increase in intracellular Ca^2+^ concentration [97]. Consequently, disruption of calcium homeostasis has profound consequences for sperm function and represents one of the key molecular mechanisms through which oxidative and inflammatory stress impair male fertility.

The CatSper (cation channel of sperm) complex constitutes the principal pathway for calcium entry in mammalian spermatozoa [97]. This sperm-specific ion channel is composed of multiple pore-forming and auxiliary subunits that cooperate to regulate calcium influx in response to physiological stimuli encountered within the female reproductive tract. Activation of CatSper initiates a cascade of downstream signaling events involving cyclic AMP (cAMP), protein kinase A (PKA), calcium/calmodulin-dependent proteins, and calcium-binding proteins such as CABYR, collectively promoting hyperactivation and directional sperm migration toward the oocyte [97,98].

Increasing evidence indicates that oxidative stress interferes with these signaling pathways at multiple levels. Reactive oxygen species can induce oxidative modifications of ion channels, signaling proteins, and calcium-binding molecules, thereby impairing intracellular calcium homeostasis. In addition, excessive ROS may alter membrane lipid composition and fluidity, indirectly affecting the activity of membrane-associated ion channels, including CatSper. These alterations compromise the finely tuned calcium oscillations required for capacitation and hyperactivation, ultimately reducing fertilization competence.

Proteomic studies further support the involvement of calcium signaling in oxidative stress-mediated infertility by identifying differential expression of proteins associated with calcium transport, calcium sensing, and phosphorylation-dependent signaling pathways. Altered abundance of proteins such as CABYR, together with dysregulation of PKA-associated signaling complexes, suggests that oxidative injury extends beyond structural damage to affect the molecular machinery coordinating intracellular signal transduction. These findings reinforce the concept that calcium signaling represents a critical molecular interface linking environmental and oxidative insults to functional sperm impairment.

Importantly, calcium signaling does not operate as an isolated pathway. Instead, it is tightly integrated with mitochondrial ATP production, redox regulation, and cytoskeletal remodeling, allowing spermatozoa to rapidly adapt their motility patterns during transit through the female reproductive tract. Consequently, disruption of calcium homeostasis has widespread downstream effects that extend beyond hyperactivation, influencing capacitation, acrosome reaction, and ultimately sperm–oocyte interaction.

Collectively, proteomic and functional evidence indicates that oxidative stress-induced dysregulation of calcium signaling contributes substantially to impaired sperm function. Rather than representing isolated alterations in individual proteins, these changes reflect coordinated disruption of signaling networks that are essential for the acquisition of fertilization competence.

### 5.3. Mitochondrial Bioenergetics and Redox Homeostasis

Mitochondria are fundamental regulators of sperm function, serving both as a major source of ATP required for motility and as important contributors to intracellular redox homeostasis. Located within the sperm midpiece, the mitochondrial sheath generates energy necessary to sustain progressive motility while simultaneously participating in the regulation of reactive oxygen species production. This dual role makes mitochondria both indispensable for normal sperm physiology and particularly vulnerable to oxidative damage. Consequently, mitochondrial dysfunction has emerged as one of the most consistent proteomic hallmarks of oxidative stress-related male infertility [92,99].

Proteomic analyses have consistently identified alterations in proteins involved in oxidative phosphorylation, electron transport chain activity, tricarboxylic acid metabolism, and ATP synthesis. Differential expression of ATP synthase subunits, cytochrome c oxidase proteins, NADH dehydrogenase subunits, and ubiquinol–cytochrome c reductase components has been reported in infertile patients, particularly those presenting with asthenozoospermia. Reduced abundance of these proteins is generally associated with impaired ATP production, decreased progressive motility, and diminished fertilization potential [94,99].

Beyond their role in energy production, mitochondria are central regulators of intracellular redox balance. Under physiological conditions, low levels of ROS generated by the electron transport chain contribute to sperm capacitation and signaling. However, excessive ROS production overwhelms antioxidant defense mechanisms, leading to oxidative damage of mitochondrial proteins, lipids, and mitochondrial DNA.

Proteomic studies have demonstrated that this imbalance is accompanied by dysregulation of antioxidant enzymes, including members of the peroxiredoxin (PRDX) family, glutathione peroxidases (GPXs), superoxide dismutase 1 (SOD1), and components of the thioredoxin system, all of which are essential for maintaining mitochondrial integrity. Among these proteins, peroxiredoxin-6 (PRDX6) has attracted particular attention because of its dual enzymatic activity as both a peroxidase and a calcium-independent phospholipase A_2_. This functional profile enables PRDX6 not only to detoxify reactive oxygen species but also to participate in the repair of oxidized membrane phospholipids, thereby preserving membrane integrity and sperm viability. Reduced expression or oxidative inactivation of PRDX6 has been associated with increased lipid peroxidation, impaired mitochondrial function, decreased sperm motility, and elevated DNA fragmentation [15].

An important concept emerging from proteomic studies is the existence of a self-perpetuating cycle linking mitochondrial dysfunction and oxidative stress. Damage to components of the electron transport chain reduces ATP production while simultaneously increasing electron leakage and ROS generation. The resulting oxidative damage further impairs mitochondrial proteins and membranes, amplifying ROS production and progressively compromising sperm function. This vicious cycle ultimately culminates in reduced motility, loss of membrane integrity, activation of apoptotic pathways, and decreased fertilization capacity.

Recent systems-level proteomic analyses further indicate that mitochondrial dysfunction cannot be considered independently of other cellular processes. Alterations in mitochondrial proteins are consistently accompanied by dysregulation of calcium signaling, antioxidant defense systems, cytoskeletal organization, and protein quality-control pathways, underscoring the extensive molecular crosstalk governing sperm physiology. Rather than representing an isolated metabolic defect, mitochondrial dysfunction should therefore be regarded as a central hub linking oxidative stress to multiple downstream alterations that collectively determine sperm quality and reproductive competence.

From a translational perspective, mitochondrial proteins constitute some of the most promising biomarker candidates identified by reproductive proteomics. Their consistent dysregulation across different causes of male infertility, together with their direct association with sperm motility and fertilization outcomes, highlights their potential utility for patient stratification, prognosis, and the development of targeted therapeutic strategies aimed at restoring mitochondrial function and redox homeostasis.

### 5.4. Membrane Remodeling, Lipid–Protein Interface, and Capacitation Competence

The acquisition of fertilization competence requires profound biochemical and structural remodeling of the sperm plasma membrane during capacitation. This highly coordinated maturation process is characterized by cholesterol efflux, redistribution of membrane lipid rafts, increased membrane fluidity, activation of phosphorylation-dependent signaling pathways, and reorganization of membrane-associated proteins. Together, these events prepare spermatozoa for hyperactivation, acrosome reaction, and ultimately sperm–oocyte fusion [91,100].

Because sperm membranes are exceptionally rich in polyunsaturated fatty acids, they are particularly susceptible to oxidative damage. Excessive ROS production promotes lipid peroxidation, resulting in altered membrane fluidity, disruption of lipid–protein interactions, and impairment of receptor-mediated signaling. These structural alterations compromise the dynamic membrane remodeling required for capacitation and represent one of the earliest functional consequences of oxidative stress in spermatozoa.

Proteomic analyses have identified numerous membrane-associated proteins that are differentially expressed or redistributed during sperm capacitation. Among the most extensively investigated are sperm adhesion molecule 1 (SPAM1), basigin (BSG/CD147), annexins, and members of the ADAM family, all of which participate in sperm migration, zona pellucida recognition, acrosome reaction, and gamete interaction [91].

Altered abundance, localization, or post-translational modification of these proteins may interfere with membrane organization and compromise the ability of spermatozoa to respond appropriately to capacitation-associated stimuli. Proteomic studies further indicate that capacitation involves coordinated remodeling of proteins implicated in motility, signaling, energy metabolism, membrane dynamics, and stress responses [91,100].

Recent evidence indicates that oxidative stress influences not only membrane proteins but also the organization of membrane microdomains that regulate intracellular signaling. Lipid raft disruption may impair the assembly of signaling complexes controlling calcium influx, protein tyrosine phosphorylation, and receptor activation, thereby affecting multiple downstream events required for fertilization. Consequently, alterations in membrane composition propagate throughout the sperm signaling network, amplifying the detrimental effects of oxidative stress.

Importantly, membrane remodeling is closely integrated with mitochondrial metabolism, calcium signaling, and antioxidant defense systems. Efficient capacitation depends on continuous communication between these pathways, enabling spermatozoa to rapidly respond to environmental cues encountered within the female reproductive tract. Oxidative stress disrupts this molecular coordination, reducing the efficiency of capacitation and limiting the ability of spermatozoa to undergo acrosome reaction and establish productive interactions with the oocyte.

Overall, reproductive proteomics has demonstrated that defective membrane remodeling represents far more than a structural abnormality. Instead, it reflects widespread disruption of interconnected signaling networks that regulate sperm activation and fertilization competence, making membrane-associated proteins attractive candidates for both biomarker discovery and therapeutic intervention.

### 5.5. Proteostasis and Stress-Response Pathways

Maintenance of protein homeostasis, or proteostasis, is essential for preserving sperm functionality throughout the journey from ejaculation to fertilization. Unlike somatic cells, mature spermatozoa possess extremely limited transcriptional and translational activity and therefore depend almost entirely on pre-existing proteins and efficient quality-control mechanisms to maintain structural integrity and functional competence. As a consequence, oxidative and inflammatory stress can have particularly severe effects on sperm physiology by promoting protein oxidation, misfolding, aggregation, and degradation.

Proteomic investigations consistently identify alterations in molecular chaperones and stress-response proteins among the characteristic signatures of infertile patients. Members of the heat shock protein family—including HSPA2 and HSP90AA1—are repeatedly reported as differentially expressed or post-translationally modified in association with male infertility and oxidative stress [4,101]. These proteins play essential roles in protein folding, stabilization of signaling complexes, prevention of protein aggregation, and regulation of sperm maturation, capacitation, and fertilization.

Interestingly, increased expression of some heat shock proteins may represent a compensatory response to oxidative injury. By enhancing chaperone activity, spermatozoa may attempt to preserve the functionality of oxidatively damaged proteins and maintain cellular homeostasis despite adverse environmental conditions. Conversely, reduced abundance or altered post-translational modification of key chaperones such as HSPA2 may contribute directly to defective sperm maturation and impaired oocyte recognition [101]. Persistent oxidative stress may eventually overwhelm these protective mechanisms, resulting in irreversible protein damage and progressive deterioration of sperm function.

In parallel, components of the ubiquitin–proteasome system (UPS) are frequently dysregulated in infertile men. The UPS is responsible for recognizing and eliminating damaged or misfolded proteins while regulating protein turnover during sperm maturation and fertilization. Impaired proteasomal activity may therefore lead to the accumulation of dysfunctional proteins, disruption of signaling pathways, and progressive loss of sperm functionality.

Systems biology approaches suggest that proteostasis should not be considered an isolated pathway but rather an integrative mechanism coordinating antioxidant defense, mitochondrial function, cytoskeletal organization, and membrane remodeling. Dysfunction of protein quality-control systems therefore amplifies the effects of oxidative stress across multiple cellular compartments, contributing to the widespread proteomic remodeling observed in male infertility.

Collectively, these findings identify proteostasis as a central regulatory node linking oxidative stress to functional sperm deterioration. The consistent dysregulation of molecular chaperones and proteasome-associated proteins further supports their potential utility as biomarkers of sperm quality and as therapeutic targets aimed at restoring cellular resilience under conditions of oxidative stress.

### 5.6. Sperm–Egg Interaction and Fertilization Competence

Successful fertilization represents the culmination of a series of highly coordinated molecular events that enable spermatozoa to recognize, bind, penetrate, and ultimately fuse with the oocyte. This process requires the precise temporal and spatial regulation of multiple proteins involved in zona pellucida recognition, acrosome reaction, membrane adhesion, and gamete fusion. Consequently, alterations affecting any of these molecular mechanisms may significantly compromise fertilization success despite apparently normal conventional semen parameters. Proteomic and functional studies have demonstrated that oxidative and inflammatory stress may alter proteins directly or indirectly involved in sperm–oocyte interaction. Among the most consistently reported are IZUMO1, SPACA1, SPACA3, SPACA4, acrosin-binding protein (ACRBP), zona pellucida-binding protein (ZPBP), and several members of the ADAM family [91]. These proteins regulate essential events ranging from acrosomal integrity and zona pellucida binding to adhesion and membrane fusion with the oocyte.

Recent evidence has expanded the molecular model of sperm–oocyte fusion beyond the classical IZUMO1–JUNO interaction. Although IZUMO1 and JUNO are essential for gamete recognition and adhesion, their interaction alone is insufficient to drive membrane fusion. DCST1 and DCST2 have emerged as evolutionarily conserved sperm factors required for the fusion step itself, since their loss prevents sperm–oocyte fusion despite preserved binding capacity [102]. Accordingly, sperm proteomic modules related to fertilization competence should include DCST1/DCST2 together with IZUMO1, SPACA family proteins, acrosomal proteins and other sperm–egg interaction molecules.

Altered expression, abnormal localization, or oxidative modification of these molecules may be associated with reduced fertilization rates and poorer reproductive outcomes. Emerging evidence indicates that many of these alterations do not occur independently but arise as downstream consequences of broader molecular disturbances affecting capacitation, calcium signaling, mitochondrial metabolism, and membrane remodeling. Because successful sperm–egg interaction requires coordinated activation of all these biological processes, oxidative stress ultimately compromises fertilization competence through cumulative disruption of multiple interconnected pathways rather than through isolated molecular defects.

Recent advances in reproductive proteomics further suggest that proteins involved in gamete recognition may represent particularly valuable biomarkers because they directly reflect the functional competence of spermatozoa. Unlike conventional semen parameters, which provide only indirect information on fertilization potential, the expression and localization of proteins such as IZUMO1 and SPACA family members offer mechanistic insight into the sperm’s capacity to complete the final stages of fertilization.

Taken together, the proteomic signatures described throughout this chapter illustrate the highly interconnected nature of sperm biology. Oxidative and inflammatory stress do not selectively affect individual proteins but instead remodel complex molecular networks controlling motility, intracellular signaling, mitochondrial bioenergetics, membrane dynamics, protein quality control, and gamete interaction. Understanding these integrated protein networks is essential for identifying robust biomarkers, improving patient stratification, and developing targeted therapeutic strategies aimed at preserving or restoring male reproductive function.

## 6. Clinical Translation: Biomarkers, Patient Stratification, and Endpoints

Conventional semen analysis remains the clinical starting point, but sperm proteomics adds the molecular resolution needed to define sperm quality more precisely by linking abnormal function to specific disrupted pathways rather than to descriptive semen features alone. In inflammatory and oxidative male infertility, proteomic profiles can uncover coordinated defects in mitochondrial function, redox control, motility structures, capacitation, and chromatin stability, which supports biologically meaningful stratification of patients and stronger translational potential than routine semen parameters alone. These molecular signatures may help distinguish patients with predominant oxidative damage, inflammatory activation, defective spermatogenesis, accessory gland dysfunction or mixed phenotypes, thereby improving mechanistic stratification and potentially guiding therapeutic decisions [4,9,56,59,70]. Inflammatory and oxidative sperm injury may be captured by different layers of information, including seminal ROS, oxidation-reduction potential, lipid peroxidation, total antioxidant capacity, sperm DNA fragmentation, cytokines and soluble immune mediators. Among these, emerging biomarkers such as suPAR and sST2 may provide additional information on inflammatory activation and tissue-remodeling pathways, particularly in conditions such as MAGI or prostatitis, where accessory gland inflammation modifies the seminal microenvironment. In this context, biomarkers should not be considered only as diagnostic tools, but also as instruments for patient stratification, treatment monitoring and endpoint definition. Their integration with sperm proteomic signatures may support a more precise classification of patients than single-marker approaches [24,53,103]. From a translational perspective, the most clinically relevant proteomic modules are those that can be linked to measurable reproductive endpoints. These may include sperm motility, sperm DNA integrity, fertilization rate, embryo development, implantation, clinical pregnancy, live birth and spontaneous conception. Although multi-marker biomarker panels and sperm proteomics show considerable promise for refining the mechanistic stratification of patients with inflammatory and oxidative male infertility, their routine clinical implementation remains premature, given the persistent lack of assay standardization, validated thresholds, and robust prospective evidence.

### 6.1. Therapeutic Implications: Antioxidants and Anti-Inflammatory Strategies Guided by Proteomics

The therapeutic use of antioxidants and anti-inflammatory strategies in male infertility remains biologically well grounded but clinically heterogeneous. Oxidative stress is a recognized contributor to sperm dysfunction, and a range of antioxidants—including vitamin C, vitamin E, coenzyme Q10, carnitines, selenium, zinc, N-acetylcysteine, lycopene, and combined formulations—has been investigated for potential effects on semen quality, oxidative damage, and sperm DNA fragmentation. However, the evidence remains variable, and any benefit appears to depend on patient selection, baseline redox status, treatment duration, formulation, dose, and the specific clinical context [104,105,106,107]. For this reason, antioxidant supplementation should not be regarded as a universal treatment for male infertility. The AUA/ASRM guideline notes that supplements, including antioxidants and vitamins, have uncertain clinical value, and that current evidence remains insufficient to support specific agents for male infertility. In contrast, the SIAMS position statement supports a more targeted use of antioxidants in idiopathic couple infertility when altered semen parameters and abnormal sperm DNA fragmentation are documented after appropriate diagnostic work-up. More recent expert guidance from the Global Andrology Forum further supports this precision-based approach, emphasizing that oxidative stress should be objectively documented before initiating antioxidant therapy and cautioning against indiscriminate or prolonged treatment because of the potential risk of reductive stress [107,108,109]. Proteomics may help reduce this therapeutic uncertainty by identifying patients with molecular profiles consistent with oxidative and redox-related sperm dysfunction, who may be more likely to respond to targeted redox-based interventions. In contrast, patients whose sperm proteome is dominated by signatures of severe spermatogenic impairment, structural flagellar abnormalities, genetic defects, or extensive chromatin remodeling may be less likely to benefit from empirical antioxidant therapy alone. From this perspective, proteomics could support the distinction between redox-responsive and non-redox-responsive infertility phenotypes. Anti-inflammatory strategies may be particularly relevant when proteomic and biochemical data indicate inflammatory activation rather than isolated oxidative stress. In conditions such as MAGI, prostatitis or leukocyte-rich semen, the therapeutic target may not be ROS alone, but the upstream inflammatory process that sustains the pro-oxidant seminal environment. In these settings, management may require microbiological evaluation, management of infection when present, modulation of accessory gland inflammation, lifestyle correction and, in selected cases, nutraceuticals with combined antioxidant and anti-inflammatory properties. Within this framework, proteomic monitoring may help clarify whether intervention is associated with restoration of sperm pathways related to motility, mitochondrial function, membrane organization and fertilization competence, rather than with changes in conventional semen parameters.

### 6.2. Linking Proteomic Modules to ART Outcomes and Spontaneous Conception

A major challenge for clinical translation is linking sperm proteomic signatures to reproductive outcomes. Several sperm proteins and pathways identified by proteomic studies have been associated with motility, capacitation, acrosome reaction, zona pellucida interaction, sperm–oocyte fusion, mitochondrial function and DNA integrity. These biological processes are directly relevant to both natural conception and ART outcomes. For example, alterations in proteins involved in axonemal organization, CatSper-associated signaling, mitochondrial oxidative phosphorylation, chaperone activity, proteasomal function and membrane remodeling may contribute to reduced fertilization competence even when standard semen parameters are only moderately impaired [8,59,70,110]. In ART, sperm proteomic profiles may provide information complementary to conventional semen analysis. ICSI can bypass some aspects of sperm number and motility, but it does not necessarily overcome defects related to sperm DNA damage, oocyte-activating capacity, centrosomal function, chromatin integrity or epigenetic/proteomic abnormalities. Therefore, proteomic markers may help explain why some couples experience poor fertilization, impaired embryo development or recurrent ART failure despite apparently acceptable semen parameters. However, robust clinical translation requires studies designed around reproductive endpoints rather than only differential protein abundance. Spontaneous conception represents an even more complex but highly relevant endpoint. Unlike ART, natural conception requires the full sequence of sperm functional competence: motility through the female reproductive tract, capacitation, hyperactivation, zona binding, acrosome reaction, oocyte fusion and contribution to early embryogenesis. Proteomic modules related to flagellar function, mitochondrial activity, membrane dynamics and sperm–egg interaction may therefore be particularly informative in identifying sperm profiles compatible with natural fertility. Longitudinal studies assessing spontaneous pregnancy after medical or nutraceutical interventions would provide a more clinically meaningful validation of sperm proteomic signatures than cross-sectional comparisons alone.

### 6.3. Feasibility: From Discovery Proteomics to Clinical Assays

Despite its biological value, discovery proteomics is not yet a routine clinical tool in andrology. High-resolution LC–MS/MS allows deep characterization of the sperm proteome, but it remains expensive, technically demanding and sensitive to pre-analytical variability, sample purity, data processing pipelines and statistical thresholds. Moreover, most proteomic studies are still limited by small sample size, heterogeneous clinical phenotypes and insufficient validation in independent cohorts. These limitations currently restrict the direct use of untargeted sperm proteomics in routine infertility work-up [59,70,111]. The translational path from discovery proteomics to clinical assays requires several steps. First, candidate proteins or proteomic modules must be consistently associated with well-defined clinical phenotypes and reproductive outcomes. Second, these candidates should be validated using targeted methods such as selected reaction monitoring, parallel reaction monitoring, ELISA, immunoblotting, immunofluorescence or flow cytometry-based assays. Third, clinically meaningful cut-offs, analytical reproducibility, intra-individual variability and added value over existing tests must be established. Only after this process can sperm proteomic markers move from discovery datasets to clinically actionable assays. A practical translational strategy may involve reducing complex proteomic signatures into focused biomarker panels. Rather than measuring hundreds of proteins, clinical assays could target representative proteins from key functional modules, such as mitochondrial metabolism, redox homeostasis, flagellar structure, membrane remodeling and sperm–egg interaction. These targeted panels could be combined with established biochemical readouts, including lipid peroxidation, oxidation-reduction potential, antioxidant capacity, sperm DNA fragmentation and inflammatory biomarkers such as cytokines, suPAR or sST2. Although the present review intentionally focuses on spermatozoa and sperm proteomics, future translational studies may benefit from integrating sperm proteomic data with complementary multi-omics approaches and artificial intelligence-based analytical tools to improve biomarker discovery, patient stratification and prediction of reproductive outcomes. Beyond the identification of individual protein markers, artificial intelligence-based approaches may be especially valuable for reading and decoding proteomic pathway signatures underlying specific pathological states, thereby improving mechanistic interpretation and the clinical usability of complex molecular data [112]. This integrated framework could preserve the biological depth of proteomics while making its application more compatible with routine clinical laboratory workflows. Feasibility also depends on sample type, since whole semen, seminal plasma and purified spermatozoa provide different information and have different translational potential. Seminal plasma biomarkers are generally easier to standardize and may be suitable for scalable immunoassays, while purified sperm proteomics provides more direct information on sperm-intrinsic molecular damage but requires stricter processing. A clinically realistic future may therefore involve a two-level approach: first-line seminal plasma and redox biomarkers for screening and stratification, followed by targeted sperm proteomic assays in selected patients with unexplained, recurrent or treatment-resistant infertility.

### 6.4. Precision Supplementation: Who Benefits and Why

Precision supplementation represents a shift from empirical antioxidant use toward mechanism-guided intervention. The key question is not whether antioxidants are broadly useful in male infertility, but which patients are most likely to benefit, which formulation is appropriate, and which endpoint should be used to define response. Current evidence and guideline positions suggest that supplementation is more justifiable when oxidative stress, sperm DNA fragmentation, abnormal semen parameters or inflammatory–oxidative phenotypes are documented, rather than in unselected infertile men [107,108,109]. The most plausible candidates are patients with documented oxidative stress and a preserved potential for functional recovery. This group may include men with idiopathic infertility and abnormal redox markers, elevated sperm DNA fragmentation, varicocele-associated oxidative stress, lifestyle-related oxidative burden, metabolic dysfunction, environmental exposure or inflammatory conditions after exclusion of active infection requiring specific treatment. In these settings, supplementation is better viewed as part of a broader management strategy that may also include correction of lifestyle factors, smoking cessation, weight management, treatment of varicocele when indicated and management of genital tract inflammation. Proteomics may help define the biological basis for supplementation by identifying molecular profiles consistent with redox-responsive sperm dysfunction. Such profiles may include altered abundance of antioxidant enzymes, mitochondrial respiratory proteins, peroxiredoxins, glutathione-related proteins, chaperones, lipid metabolism proteins, and flagellar proteins vulnerable to oxidative injury. Treatment-associated improvement may, in turn, be reflected by restoration of proteins linked to motility, mitochondrial function, and membrane remodeling, together with reduced stress-response and extracellular inflammatory signatures. In this framework, proteomics could serve both as a pre-treatment stratification tool and as a molecular endpoint for response assessment. At the same time, precision supplementation requires caution. Excessive or inappropriate antioxidant exposure may blunt the physiological ROS signaling required for capacitation, hyperactivation, and the acrosome reaction, thereby creating a risk of reductive stress. This point is critical because sperm function depends on maintenance of a narrow redox range rather than on indiscriminate ROS suppression. Accordingly, supplementation should be biologically justified, time-bounded, and evaluated against appropriate endpoints, with the aim of restoring redox homeostasis rather than achieving antioxidant excess [1,107,113]. Overall, the most defensible clinical translation is not the empirical use of antioxidants in male infertility, but the application of targeted redox and anti-inflammatory interventions in biologically defined patient subgroups. Within this model, sperm proteomics may support this transition by identifying molecular signatures of injury, defining response-related pathways and providing clinically meaningful endpoints for treatment monitoring.

## 7. Conclusions

Inflammatory and oxidative injury in male infertility represents a complex biological continuum in which local and systemic insults converge on interconnected molecular networks governing sperm function. Proteomics has substantially expanded our understanding of this complexity, revealing coordinated alterations in flagellar architecture, calcium signaling, mitochondrial bioenergetics, redox homeostasis, membrane remodeling, proteostasis, and fertilization competence that remain largely invisible to conventional semen analysis. However, translating these molecular signatures into clinically meaningful tools requires rigorous control of pre-analytical variables, well-defined patient phenotypes, standardized analytical workflows, and validation against relevant reproductive outcomes. Rather than replacing established diagnostic approaches, sperm proteomics may provide an additional molecular layer capable of refining patient stratification and identifying biologically distinct, potentially treatment-responsive phenotypes. Future integration of proteomic signatures with redox and inflammatory biomarkers, clinical data, and advanced computational approaches may ultimately support a shift from empirical management toward mechanism-based precision andrology.

## Figures and Tables

**Table 2:** The table summarizes the main proteins and protein families discussed across the functional modules reported in Figure 3, including flagellar architecture and motility, Ca^2+^ signaling, mitochondrial bioenergetics, redox homeostasis, membrane remodeling, proteostasis, and sperm–oocyte interaction. For each candidate, the reported direction or type of alteration, associated infertility phenotype or clinical context, current level of evidence, and available validation methods are indicated. This overview highlights the distinction between discovery-stage proteomic candidates and proteins supported by targeted validation or functional evidence.

Protein/Protein Family	Functional Module	Reported Alteration	Associated Phenotype/Context	Current Evidence Level	Validation Method Reported
**AKAP4**	Flagellar architecture and motility	Reduced abundance/altered expression	Asthenozoospermia, impaired progressive motility, reduced fertilization potential	Recurrent discovery proteomics; candidate biomarker	Mainly LC–MS/MS; WB reported in some studies
**AKAP3**	Flagellar architecture and motility	Altered abundance	Impaired capacitation and flagellar function	Discovery/early validation	LC–MS/MS; targeted validation variable
**ODF1**	Flagellar architecture and motility	Reduced or altered abundance	Defective flagellar structure, impaired motility	Discovery-stage candidate	LC–MS/MS
**ODF2**	Flagellar architecture and motility	Reduced abundance	Asthenozoospermia; chronic inflammatory/MAGI phenotypes	Discovery plus targeted validation in selected cohorts	LC–MS/MS; IF in selected studies
**CABYR**	Flagellar architecture/Ca^2+^ signaling	Altered abundance	Impaired motility, defective hyperactivation/capacitation	Discovery-stage candidate	LC–MS/MS
**CatSper complex proteins**	Ca^2+^ signaling and hyperactivation	Altered abundance or inferred pathway dysregulation	Impaired calcium influx, hyperactivation defects, reduced fertilization competence	Strong functional evidence; proteomic detection variable	Functional/genetic studies; proteomics in selected datasets
**ATP5A1/ATP5B**	Mitochondrial bioenergetics	Reduced abundance	Asthenozoospermia, impaired ATP production	Recurrent discovery proteomics	LC–MS/MS; WB in selected studies
**COX proteins**	Mitochondrial bioenergetics	Altered/reduced abundance	Mitochondrial dysfunction, impaired motility	Discovery-stage candidate	LC–MS/MS
**NADH dehydrogenase subunits, complex I**	Mitochondrial bioenergetics	Altered/reduced abundance	Reduced oxidative phosphorylation, asthenozoospermia	Discovery-stage candidate	LC–MS/MS
**Ubiquinol–cytochrome c reductase components, complex III**	Mitochondrial bioenergetics	Altered abundance	Impaired mitochondrial function and redox imbalance	Discovery-stage candidate	LC–MS/MS
**PRDX family proteins**	Redox homeostasis	Altered abundance and/or oxidative modification	Oxidative stress, lipid peroxidation, impaired sperm function	Recurrent discovery plus redox-focused evidence	LC–MS/MS; redox/PTM-directed assays in selected studies
**PRDX6**	Redox homeostasis/membrane protection	Reduced abundance or oxidative inactivation should be considered distinct mechanisms	Increased lipid peroxidation, impaired motility, DNA fragmentation	Functional and redox-focused evidence	LC–MS/MS; enzymatic/redox assays; targeted validation in selected studies
**GPXs**	Redox homeostasis	Altered abundance	Oxidative stress, impaired antioxidant defense	Discovery-stage/pathway-level evidence	LC–MS/MS
**SOD1**	Redox homeostasis	Altered abundance	Impaired antioxidant defense, oxidative sperm injury	Discovery-stage/pathway-level evidence	LC–MS/MS
**SPAM1**	Membrane remodeling and sperm–oocyte interaction	Altered abundance/localization	Impaired zona binding, migration, fertilization competence	Discovery plus functional plausibility	LC–MS/MS; localization studies in selected contexts
**BSG/CD147**	Membrane remodeling	Altered abundance	Altered membrane organization and capacitation competence	Discovery-stage candidate	LC–MS/MS
**Annexins**	Membrane remodeling/lipid–protein interface	Altered abundance	Membrane remodeling defects, capacitation impairment	Discovery-stage candidate	LC–MS/MS
**ADAM family proteins**	Membrane remodeling/sperm–egg interaction	Altered abundance	Gamete interaction defects, fertilization impairment	Discovery plus functional evidence for family members	LC–MS/MS; functional studies variable
**HSPA2**	Proteostasis/chaperones	Reduced abundance or altered PTM	Defective sperm maturation, capacitation, oocyte recognition	Recurrent candidate with functional support	LC–MS/MS; WB/IF in selected studies
**HSP90/HSP90AA1**	Proteostasis/chaperones	Altered abundance or PTM	Oxidative stress response, impaired protein folding/signaling	Discovery-stage/early validation	LC–MS/MS; targeted validation variable
**UPS-related proteins**	Ubiquitin–proteasome system	Altered abundance/pathway dysregulation	Accumulation of damaged proteins, impaired maturation and fertilization	Discovery/pathway-level evidence	LC–MS/MS; functional proteasome assays in selected studies
**IZUMO1**	Sperm–egg interaction/fusion	Altered abundance/localization	Impaired gamete recognition and adhesion	Strong functional evidence; proteomic candidate	Functional studies; IF/localization; proteomics in selected datasets
**SPACA1/SPACA3/SPACA4**	Acrosomal integrity and sperm–egg interaction	Altered abundance	Defective acrosome reaction, impaired fertilization competence	Discovery-stage with functional plausibility	LC–MS/MS
**ACRBP**	Acrosomal function	Altered abundance	Acrosomal dysfunction, impaired zona interaction	Discovery-stage candidate	LC–MS/MS
**ZPBP**	Zona pellucida binding/acrosome	Altered abundance	Defective zona binding and fertilization competence	Discovery-stage candidate	LC–MS/MS
**DCST1/DCST2**	Gamete fusion machinery	Loss-of-function impairs fusion despite preserved binding; abundance data still limited	Sperm–oocyte fusion failure	Strong functional/genetic evidence; proteomic clinical validation still limited	Functional/genetic studies; proteomic validation not yet established

## Data Availability

No new data were created or analyzed in this study. Data sharing is not applicable to this article.

## Abbreviations

The following abbreviations are used in this manuscript:

ACRBP	acrosin-binding protein;
ADAM	A disintegrin and metalloproteinase;
AKAP3	A-kinase anchoring protein 3;
AKAP4	A-kinase anchoring protein 4;
ART	assisted reproductive technology;
BSG/CD147	basigin/cluster of differentiation 147;
CABYR	calcium-binding tyrosine phosphorylation-regulated protein;
CatSper	cation channel of sperm;
COX	cytochrome c oxidase;
DDA	data-dependent acquisition;
DIA	data-independent acquisition;
FASP	filter-aided sample preparation;
GPX	glutathione peroxidase;
HNE	4-hydroxynonenal;
HSPA2	heat shock protein family A member 2;
HSP90	heat shock protein 90;
HSP90AA1	heat shock protein 90 alpha family class A member 1;
IF	immunofluorescence;
IL	interleukin;
iTRAQ	isobaric tags for relative and absolute quantitation;
LC–MS/MS	liquid chromatography coupled with tandem mass spectrometry;
LFQ	label-free quantification;
MAGI	male accessory gland inflammation/infection;
MRM	multiple reaction monitoring;
NADH	nicotinamide adenine dinucleotide reduced form;
ODF1	outer dense fiber protein 1;
ODF2	outer dense fiber protein 2;
ORP	oxidation-reduction potential;
PKA	protein kinase A;
PRDX	peroxiredoxin;
PRDX6	peroxiredoxin 6;
PRM	parallel reaction monitoring;
PTM	post-translational modification;
ROS	reactive oxygen species;
SOD1	superoxide dismutase 1;
SP3	single-pot solid-phase-enhanced sample preparation;
SPACA	sperm acrosome-associated protein;
SPAM1	sperm adhesion molecule 1;
SRM	selected reaction monitoring;
sST2	soluble suppression of tumorigenicity 2;
S-Trap	suspension trapping;
suPAR	soluble urokinase plasminogen activator receptor;
TMT	tandem mass tag;
TNF-α	tumor necrosis factor alpha;
TRUS	transrectal ultrasound;
UPS	ubiquitin–proteasome system;
ZPBP	zona pellucida-binding protein
